# UCA1 lncRNA regulates γ-globin expression by modulating the miR-148b/BCL11A axis

**DOI:** 10.26508/lsa.202603620

**Published:** 2026-06-29

**Authors:** Motiur Rahaman, Shatarupa Bhattacharya, Mandrita Mukherjee, Chiranjib Bhowmick, Praphulla Chandra Shukla, Tuphan Kanti Dolai, Nishant Chakravorty

**Affiliations:** 1 School of Medical Science and Technology, IIT Kharagpur, Kharagpur, India; 2 Department of Hematology, Nil Ratan Sircar Medical College and Hospital , Kolkata, India

## Abstract

The switch from fetal to adult hemoglobin is tightly regulated during erythropoiesis, and its dysregulation can contribute to β-hemoglobinopathies. Although transcriptional repression of γ-globin by BCL11A is well established, the posttranscriptional mechanisms that sustain BCL11A expression in adult erythroid cells remain incompletely understood. Here, we report that the long noncoding RNA UCA1/miR-148b–mediated regulatory axis is critical for globin gene switching. We identified miR-148b as a direct posttranscriptional regulator of BCL11A. lncRNA UCA1, which is abundantly expressed in adult erythroid cells, functions as a molecular decoy for miR-148b, thereby attenuating miR-148b–mediated repression of BCL11A. Depletion of UCA1 increases miR-148b availability and reduces BCL11A expression, which can lead to robust induction of γ-globin. Conversely, the ectopic expression of UCA1 restores BCL11A levels by antagonizing miR-148b, thereby promoting γ-globin silencing in adult erythroid cells. Mechanistically, UCA1 orchestrates a posttranscriptional regulatory axis that reinforces γ-globin silencing by stabilizing BCL11A levels in adult erythroid cells. Taken together, our studies uncover a previously unrecognized lncRNA-mediated mechanism that integrates miRNA activity with transcriptional control to fine-tune hemoglobin switching during adult erythropoiesis.

## Abstract (precis)

The study identifies a UCA1/miR-148b–mediated regulatory axis that modulates fetal hemoglobin expression in adult erythroid cells, highlighting a potential RNA-based therapeutic strategy for β-hemoglobinopathies.

## Introduction

Fetal hemoglobin (HbF, α_2_γ_2_) is the predominant form of hemoglobin at birth. The switch from γ-globin to β-globin gene expression occurs during ontogeny, leading to the gradual replacement of fetal hemoglobin (HbF) by adult hemoglobin (HbA) (1). The complex regulatory mechanisms underlying globin gene switching are crucial to our understanding of higher eukaryotic transcriptional control. In healthy adults, HbF typically constitutes less than 1% of total hemoglobin and has limited physiological relevance (2). However, congenital, acquired, and drug-mediated strategies for HbF induction are reported to ameliorate the clinical manifestations of β-hemoglobinopathies, such as sickle cell anemia and β-thalassemia, by reducing the α-globin chain precipitation and providing an alternate form of functional hemoglobin (3, 4, 5). Therefore, unraveling the mechanisms of γ-globin gene regulation holds direct translational relevance for developing new therapies in β-hemoglobinopathies (6). Both genetic and epigenetic factors are known to influence γ-globin expression in adult erythroid cells. Naturally occurring conditions such as hereditary persistence of fetal hemoglobin, δβ-thalassemia, and pathological disorders like juvenile myelomonocytic leukemia offer valuable insights into adult γ-globin regulation (7, 8, 9). Nevertheless, a detailed understanding regarding the mechanisms to elevate γ-globin expression remains incomplete and continues to attract considerable scientific attention.

As efforts to elevate HbF levels intensify, growing evidence highlights the critical role of noncoding RNAs (ncRNAs) in modulating globin gene expression. These regulatory RNAs offer promising strategies for reactivating γ-globin genes in adult erythroid cells (10). Among these, long noncoding RNAs (lncRNAs)—a major class of ncRNAs with transcript length longer than 200 nucleotides—have emerged as major focus of translational research in the recent decades. Once dismissed as transcriptional noise, lncRNAs are now recognized for their regulatory functions at transcriptional, posttranscriptional, and translational levels. They can modulate processes such as chromatin remodeling, nuclear architecture, cellular differentiation, and lineage commitment (11). Over the past decade, numerous lncRNAs have been identified in erythroid cells, with several reported to regulate hematopoiesis, erythropoiesis, and red blood cell maturation (12, 13, 14). Apart from lncRNAs, microRNAs (miRNAs) represent a prominent class of ncRNAs that play essential roles in hematopoiesis (15). These small (∼22 nucleotide) transcripts regulate gene expression at the posttranscriptional level through mRNA degradation or translational repression (16). In our previous study, we identified disease-associated miRNA dysregulation in HbE/β-thalassemia relative to the healthy control, providing a framework for investigating the role of ncRNA-mediated regulatory networks in HbF regulation (17). Building upon these preliminary findings, the present study attempted to investigate the mechanistic role of ncRNA-mediated interactions in globin gene regulation. Previously, it has been shown that lncRNAs can function as miRNA decoys or sponges, thereby modulating the availability of miRNAs to their target transcripts and influencing diverse biological processes (18). Such competitive interactions may occur through direct interaction with the miRNA itself or with the 3′ untranslated region (3′ UTR) of target mRNAs, thereby mitigating miRNA-mediated silencing (19, 20). Recent studies have further highlighted the emerging roles of lncRNAs in HbF regulation, providing cues for HbF reactivation through ncRNA-mediated networks (21, 22). Therefore, identifying novel lncRNA-mediated regulatory mechanisms governing globin gene switching may provide promising therapeutic avenues for β-hemoglobinopathies. Genome-wide association studies have identified three key loci—*HBB*, the *HBS1L-MYB* intergenic region, and *BCL11A*—that collectively account for ∼20–45% of HbF variation across populations (23, 24, 25). In the *HBS1L-MYB* locus, a 3-bp deletion polymorphism within an enhancer gives rise to a 1,283-bp transcript termed HMI-lncRNA. Knockdown of this lncRNA results in a dramatic (∼200-fold) increase in γ-globin expression. Although the precise binding targets and downstream pathways remain unclear, HMI-lncRNA is hypothesized to interact with the *MYB* promoter, thereby modulating HbF levels (26). These findings underscore the critical role of lncRNAs in orchestrating fetal-to-adult globin switching.

Despite erythroid-specific abundance and functional relevance, the full spectrum of lncRNA-mediated regulatory mechanisms governing globin gene expression remains largely unexplored. The present study systematically investigates the lncRNA-mediated sponge regulatory network in γ-globin gene expression. By integrating sequence-based prediction with expression profiling in adult erythroid cells, we identified UCA1 as a putative miR-148b sponge. Functional validation in HUDEP-2 and CD34^+^ hematopoietic stem and progenitor cells (HSPCs) shows that UCA1 negatively regulates γ-globin expression through sequestration of miR-148b, without impeding erythroid differentiation. To our knowledge, this is the first report of UCA1 acting as a miRNA sponge to modulate γ-globin expression. Importantly, our studies provide mechanistic insights into the lncRNA/miRNA/γ-globin axis and suggest that fine-tuning the lncRNA-mediated sponge mechanisms may represent a promising therapeutic strategy for fetal hemoglobin induction in β-hemoglobinopathies.

## Results

### Prediction of sponge lncRNAs associated with γ-globin regulation

**Figure 1.**
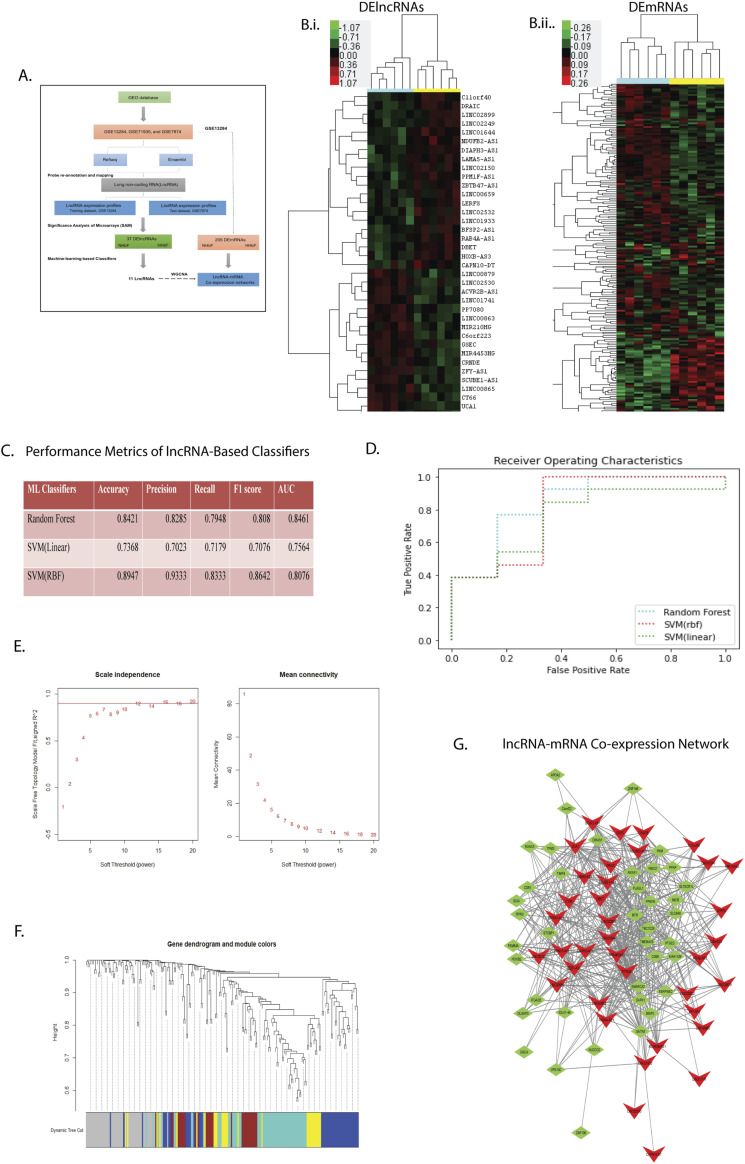
Integrated computational pipeline for identifying lncRNAs associated with γ-globin regulation. **(A)** Schematic overview of the in silico pipeline used to construct lncRNA-based classifiers for identifying high-fetal hemoglobin (HbF) conditions. The workflow includes data acquisition, preprocessing, differential expression analysis, feature selection, model training using machine learning algorithms, and classifier evaluation. **(B)** (i) Heatmap showing differentially expressed lncRNAs (DElncRNAs) between high-HbF and normal-HbF conditions in GSE13284. (ii) Heatmap showing differentially expressed mRNAs (DEmRNAs) between high-HbF and normal-HbF conditions in the same dataset. Red indicates high expression, and green indicates low expression. Bar colors denote sample type: yellow, high HbF; blue, normal HbF. **(C)** Performance matrix of different lncRNA-based machine learning (ML) classifiers for distinguishing high-HbF from low-HbF conditions. **(D)** Receiver operating characteristic curves showing the performance of lncRNA-based classifiers in distinguishing high- and low-HbF conditions in the discovery cohort. **(E)** WGCNA plots showing scale independence and mean connectivity used to determine the optimal soft-thresholding power. **(F)** Hierarchical clustering dendrogram of genes generated using adjacency-based dissimilarity, showing gene module organization. A total of five co-expression modules (blue, brown, gray, turquoise, and yellow) were identified through the analysis. **(G)** Representative color module (blue network module) is shown. Network analysis indicates that UCA1 is significantly enriched within this module. Source data are available for this figure.

Sponge long noncoding RNAs are a distinct class of gene expression regulators that, unlike transcription factors, exert their function by sharing common microRNA (miRNA) binding sites, thereby modulating the availability of miRNAs to target mRNAs. The efficacy of this interaction is influenced by the intracellular stoichiometry of miRNAs and their competing RNA targets. To identify sponge long noncoding RNAs potentially regulating γ-globin–associated genes, we developed a computational pipeline to extract lncRNA signatures from microarray datasets by repurposing Affymetrix Exon Array probes. Although these datasets were not originally designed to capture lncRNA expression, valuable information can be retrieved through probe reannotation. Using RefSeq and Ensembl annotations, we repurposed the probes and identified a total of 2,248 unique lncRNAs. A schematic overview of the computational workflow is presented in Fig 1A. Of all these, 37 lncRNAs (20 up-regulated, 17 down-regulated) were found to be significantly associated with fetal hemoglobin (HbF) regulation. Heatmaps of differentially expressed lncRNAs and mRNAs from GSE13284 are shown in Fig 1B. The complete list of 37 differentially expressed lncRNAs is provided in Supplemental information (Table S1). Next, all these 37 lncRNAs were subjected to further refinement using a supervised machine learning approach. To assess predictive performance, we compared the performance of three standard classifiers—Random Forest, SVM with linear kernel (SVM-L), and SVM with RBF kernel (SVM-RBF). Among these, the SVM-RBF classifier exhibited the highest performance, achieving an accuracy of 89.47% and a precision of 93.33%, and was therefore selected for further analysis (Fig 1C). The performance of each classifier was evaluated using receiver operating characteristic (ROC) curve analysis, as shown in Fig 1D. The analysis identified 11 lncRNAs (C6orf223, AP000679.1, GSEC, UCA1, CT66, MIR4453HG, AL135925.1, ZEB1-AS1, AC091057.1, PPM1F-AS1, and LINC02249) as the optimal set for predicting high-HbF conditions. These selected lncRNAs were subjected to further analysis.

Table S1. List of differentially expressed lncRNAs identified from normalized data using the Significance Analysis of Microarrays (SAM).

### Identification of novel lncRNA modules

To construct lncRNA-based gene regulatory networks associated with γ-globin expression, we performed weighted gene co-expression network analysis (WGCNA). A soft-thresholding power of 12 was selected for network construction, obtaining a scale-free topology fit index of 0.85 (Fig 1E). The network approximates a scale-free topology, which is consistent with the real biological network state. A total of five co-expression modules (blue, brown, gray, turquoise, and yellow) were obtained from the study (Fig 1F). Each color module comprised lncRNAs co-expressed with a set of differentially expressed protein-coding genes involved in fetal hemoglobin (HbF) regulation except for the gray module. Therefore, subsequent analysis was not performed on gray module. Notably, several lncRNAs, including UCA1, GSEC, ZEB1-AS1, and MIR4453HG, were consistently enriched across multiple modules (blue, brown, turquoise, and yellow), suggesting their critical role in the regulatory network. These lncRNAs were prioritized for experimental validation. Among them, UCA1 emerged as a prominent hub gene, exhibiting the highest number of predicted interactions, thereby indicating its prominent role as a potential sponge regulator in the γ-globin regulatory network. To further visualize the lncRNA-mRNA interactions, module-specific interaction networks were constructed using the CytoHubba plugin in Cytoscape and a representative network is shown in Fig 1G. Taken together, the data from WGCNA and network topology analysis highlight the functional relevance of these candidate lncRNAs in modulating γ-globin expression through ceRNA-mediated mechanisms, suggesting their validation in vitro.

### Functional annotation of lncRNA-associated genes

**Figure S1.**
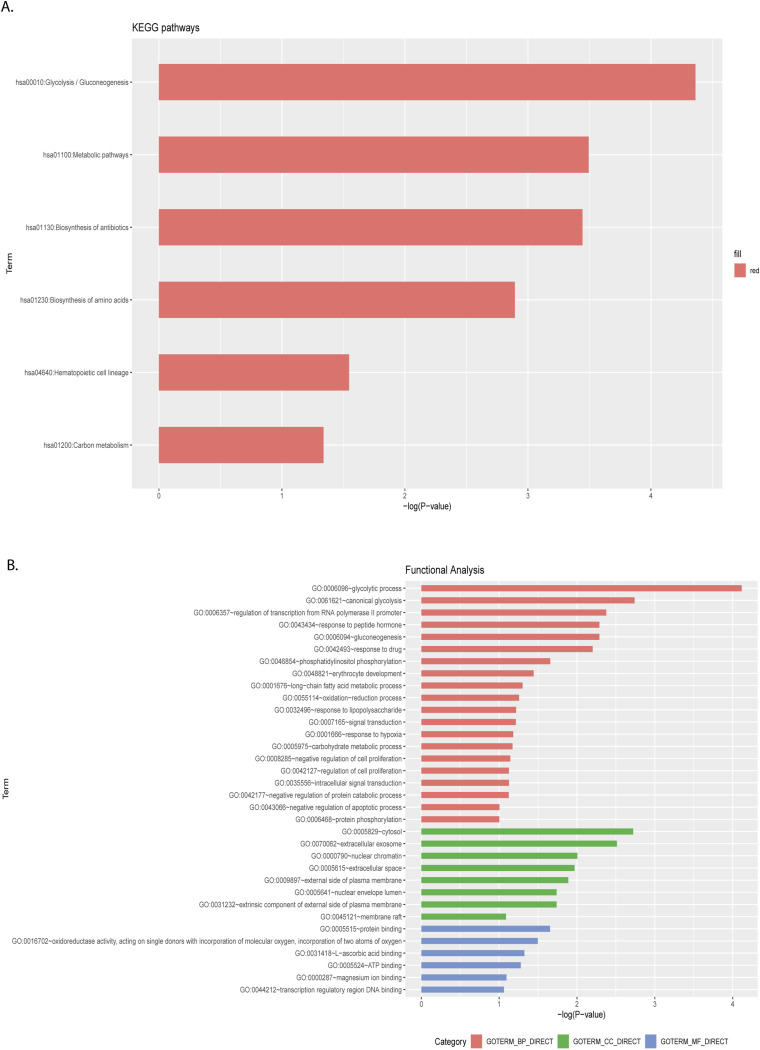
GO-term and functional enrichment analyses. **(A)** KEGG pathway analysis was performed in differentially expressed mRNAs correlated with 11 lncRNAs. The six significant (*P* < 0.05) signaling pathways are shown. All values are negative log–transformed. **(B)** Gene Ontology (GO) annotation of differentially expressed mRNAs correlated with 11 lncRNAs, with a list of the top enriched genes (*P* < 0.05), covering the domain of biological processes, cellular components, and molecular functions. Enrichment values were −log_10_ (*P*-value)-transformed.

To explore the functional relevance of lncRNA-associated gene networks, we performed Gene Ontology (GO) and pathway enrichment analyses on genes co-expressed with candidate lncRNAs identified through WGCNA. GO enrichment analysis revealed that these genes were significantly associated with biological processes related to erythrocyte development (GO: 0048821), highlighting their potential role in erythropoiesis. In terms of cellular components, cytosol (GO: 0005829) emerged as the most significantly enriched category, whereas protein binding (GO: 0005515) was the predominant molecular function. Furthermore, Kyoto Encyclopedia of Genes and Genomes pathway analysis identified significant enrichment in pathways such as hematopoietic cell lineage (hsa04640) and metabolic pathways (hsa01100). Collectively, these findings suggest that lncRNA-associated gene networks are functionally linked to erythroid development (Fig S1), and may play critical roles in globin gene regulation.

### Experimental validation of lncRNA-based regulatory networks in an in vitro model of high HbF

**Figure 2.**
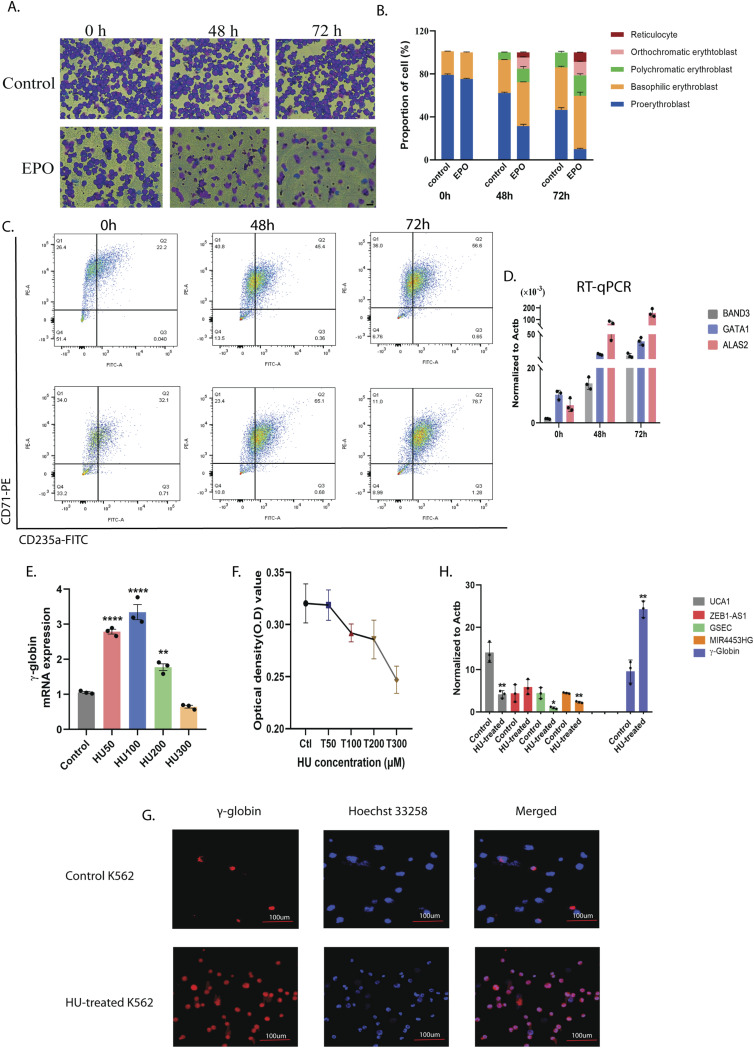
Experimental validation of key lncRNAs predicted from in silico analysis. **(A)** Representative images of May–Grünwald–Giemsa-stained cytospins showing morphological changes in K562 cells after erythropoietin (EPO; 3 IU/ml) treatment at different time points (objective lens, 20×). The scale bar indicates 20 µm. **(B)** Bar plots showing the proportions of erythroid lineage cells after erythropoietin (EPO; 3 IU/ml) treatment, based on morphological quantification. Extrapolated numbers of erythroid cell types at different time points during differentiation are presented as the mean ± SD (n = 3). **(C)** Representative flow cytometry plots showing the proportions of CD71^+^/CD235a^+^ erythroid cells at different time points (0–72 h) after erythropoietin (EPO; 3 IU/ml) treatment. Data are presented as the mean ± SD (n = 3). **(D)** RT–qPCR analysis showing expression levels of key markers of erythroid differentiation, BAND3*, *GATA1, and ALAS2. **(E)** RT–qPCR analysis showing expression levels of γ-globin in K562 cells treated with increasing concentrations (50–300 µM) of hydroxyurea (HU) drug. **(F)** Cell viability assay results in K562 cells after HU treatments. The x-axis represents drug concentrations, whereas the y-axis represents the absorbance value obtained from the MTT assay. **(G)** Representative fluorescence image showing up-regulation of γ-globin post-HU treatment (100 μM) in comparison with the nontreated control. Images were acquired using a 20× objective lens. Scale bar = 100 μm. **(H)** RT–qPCR analysis showing expression of different predicted lncRNAs in K562 cells post-HU treatment in comparison with the nontreated control. Data are representative of three independent biological replicates, and graphical data are represented as the mean ± SD. *P < 0.05, **P < 0.01, ***P < 0.001, ****P < 0.0001 by an unpaired two-tailed *t* test.

To establish a high-fetal hemoglobin (HbF)–expressing erythroid model, K562 cells were induced to undergo erythroid differentiation using erythropoietin (EPO) for 72 h, followed by hydroxyurea (HU) treatment to maximize HbF induction, as previously reported (27). Initially, erythroid differentiation over time was evaluated by May–Grünwald–Giemsa (MGG) staining (Fig 2A), and morphological analysis showed a progressive distribution of cells across distinct erythroid maturation stages, consistent with erythroid differentiation (Fig 2B). Flow cytometry analysis of EPO-treated K562 cells revealed a steady increase in CD71^+^/CD235a^+^ populations over time (0, 48, and 72 h), indicating successful erythroid differentiation. Notably, 78.7% of EPO-treated cells exhibited a CD71^+^/CD235a^+^ phenotype at 72 h, compared with 56.6% in untreated controls (Fig 2C). RT–qPCR analysis of erythroid marker genes (*BAND3*, *GATA1*, and *ALAS2*) further confirmed the differentiation (Fig 2D). Next, we sought to induce γ-globin expression by treating the differentiated cells with hydroxyurea (HU).

**Figure S2.**
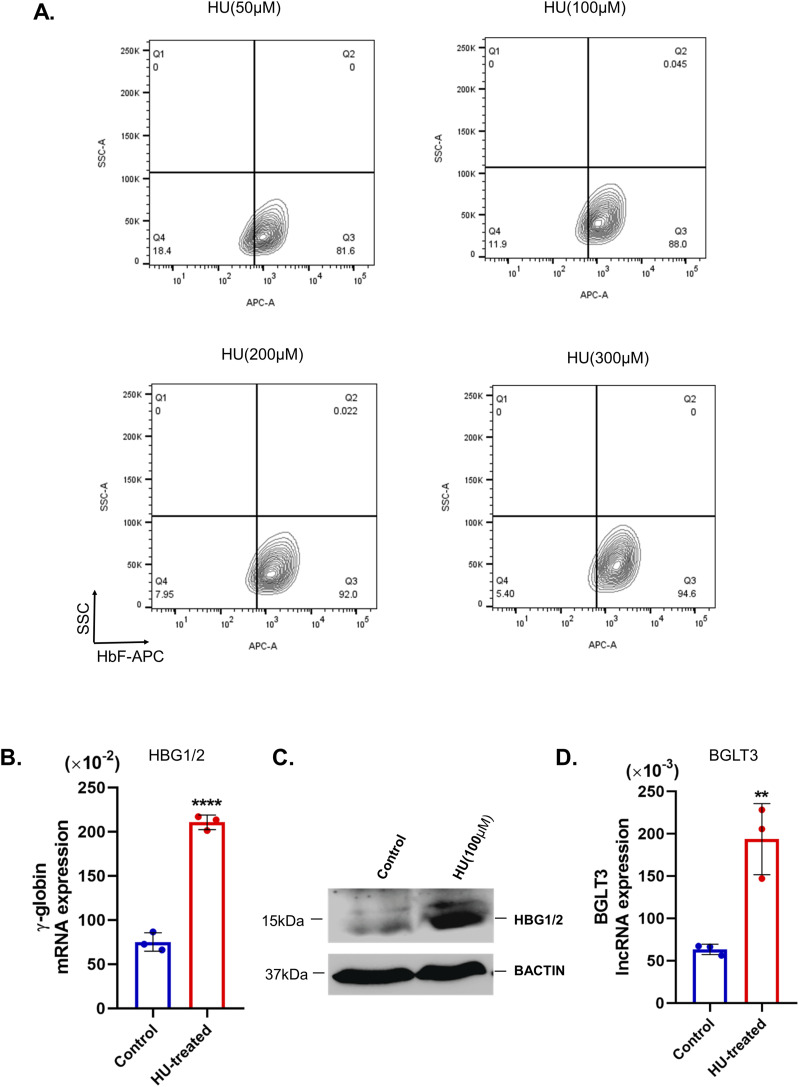
Expression of γ-globin and BGLT3 following hydroxyurea treatment in differentiated K562 cells (day 3). **(A)** Representative HbF cell staining flow cytometry plots of K562 cells treated with different concentrations (100–300 μM) of hydroxyurea (HU). **(B)** Gamma globin expression in control and HU-treated K562 cells as determined by RT–qPCR. **(C)** Immunoblot analysis with indicated antibodies of cell lysates from control and HU-treated K562 cells. Beta-actin served as a loading control. **(D)** BGLT3 expression in control and HU-treated K562 cells as determined by RT–qPCR. Data are shown as the mean ± SD of n = 3 independent experiments. Two-tailed *t* test, *P < 0.05, **P < 0.01, ***P < 0.001, ****P < 0.0001.

To optimize dosage, differentiated K562 cells were treated with increasing concentrations (50–300 μM) of the drug. RT–qPCR analysis at 24 h posttreatment revealed a dose-dependent increase in γ-globin expression, which peaked at 100 µM and declined at higher concentrations (Fig 2E), which further validated through flow cytometry (Fig S2A). In parallel, cell viability decreased significantly beyond 100 µM HU (Fig 2F), establishing 100 µM as the optimal concentration. Immunofluorescence analysis demonstrated a marked increase in γ-globin expression at this dose (Fig 2G), consistent with RT–qPCR and immunoblot results (Fig S2B and C), collectively validating effective γ-globin induction. Once the high-HbF condition was established, we first examined the expression of BGLT3, a known erythroid-specific lncRNA transcribed downstream of *HBG1* and has previously been reported to positively regulate γ-globin expression (21). In HU-treated cells, BGLT3 exhibited a coordinated expression pattern with γ-globin, where its up-regulation paralleled with γ-globin induction (Fig S2D). Next, we proceeded to validate the expression of the predicted lncRNAs identified through our in silico analysis. RT–qPCR analysis of predicted lncRNAs revealed significant down-regulation of UCA1, GSEC, and MIR4453HG in high-HbF conditions, consistent with the predictions (Fig 2H). Although ZEB1-AS1 was predicted to be down-regulated, no significant change was observed experimentally, which may reflect limitations of the predictive model. Given its high network connectivity and consistent down-regulation, UCA1 was prioritized in our study for subsequent sponge network construction and functional characterization.

### Coordinated expression of UCA1 and miR-148b elevates γ-globin expression in HbE/β-thalassemia patients

**Figure 3.**
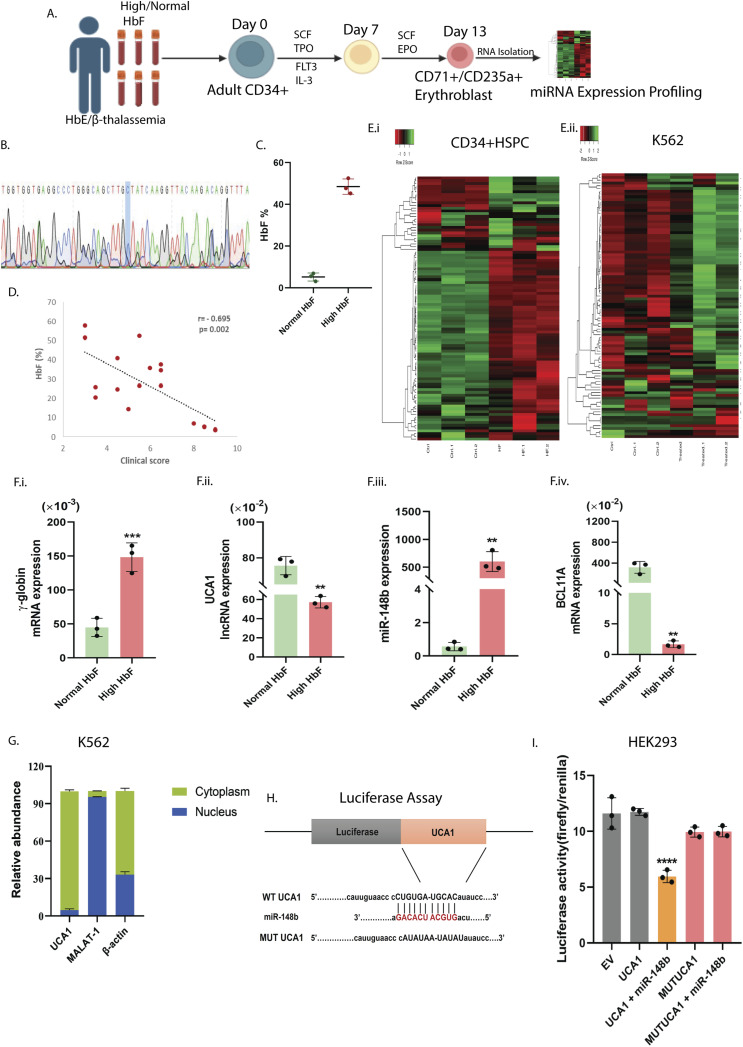
Integrated analysis of miRNA-lncRNA interactions and clinical correlations in CD34^+^ -derived erythroblasts collected from HbE/β-thalassemia patients. **(A)** Schematic overview showing miRNA expression profiling from adult CD34^+^ HSPCs obtained from HbE/β-thalassemia patients at the indicated time point. **(B)** Representative chromatogram of DNA sequences denoting HBB mutation in patient sample. Direct sequencing analysis revealed the presence of IVS-1-5(G>C) mutation in intron 1 of the β-globin gene. The blue box marked the position of the substitution in the β-globin gene. **(C)** Comparison of HbF levels between two groups, patients with normal HbF (<8%) and patients with high HbF (>40%) are shown. **(D)** Data are showing associations between HbF levels and clinical score of HbE/β-thalassemia patients. Pearson’s correlation coefficients were used to evaluate the associations of HbF levels with disease severity, which is represented as a clinical score. The Pearson correlation coefficient (r) and *P*-value are shown. **(E)** Heatmap showing differentially expressed miRNAs in (i) CD34^+^ HSPC-derived primary erythroblasts from high-HbF (n = 3) and normal-HbF (n = 3) patients at day 13 of differentiation, and (ii) HU-treated (high HbF) versus control K562 cells at day 3 of differentiation. **(F)** Expression of (i) γ-globin, (ii) UCA1, (iii) miR-148b, and (iv) BCL11A in CD34^+^ HSPC-derived primary erythroblasts from patients with high HbF (n = 3) and normal HbF (n = 3) at day 13 of differentiation, measured by RT–qPCR. **(G)** Relative abundance of UCA1 in cytoplasmic and nuclear fractions of K562 cells after differentiation (day 3). MALAT1 was used as a nuclear control, and β-actin was used as a cytoplasmic control. **(H)** Schematic workflow showing the sequence of UCA1 with a putative binding site of miR-148b. MUTUCA1 refers to a mutant variant of UCA1 in which the miR-148b binding sites have been disrupted. **(I)** Representative figure showing luciferase assay to confirm direct interaction between UCA1 and miR-148b in HEK293 cells. Results are presented as the mean ± SD (n = 3 independent experiments), unless otherwise stated. *P < 0.05, **P < 0.01, ***P < 0.001, ****P < 0.0001 by an unpaired two-tailed *t* test.

**Figure S3.**
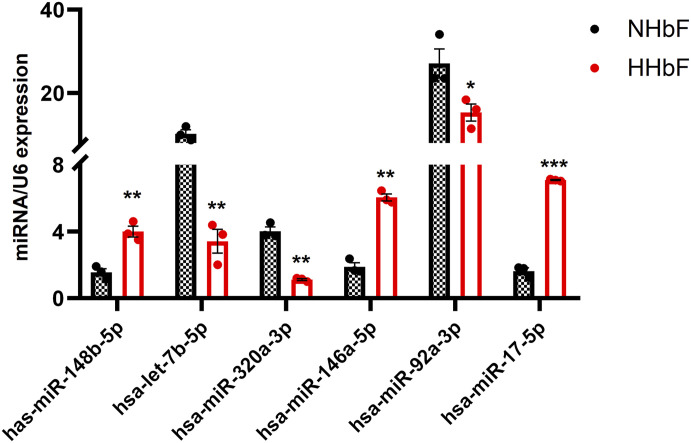
RT–qPCR-based validation of selected differentially expressed miRNAs identified from the miRNA array was performed using an independent cohort comprising high-HbF patients (HHbF, n = 3) and normal-HbF patients (NHbF, n = 3). As a reference, HbF-associated miRNA control, hsa-miR-17-5p, previously reported to be differentially expressed between HHbF and NHbF groups, was also included. Data are representative of three independent biological replicates, and graphical data are represented as the mean ± SD. *P < 0.05, **P < 0.01, ***P < 0.001, ****P < 0.0001 by an unpaired two-tailed *t* test.

**Figure S4.**
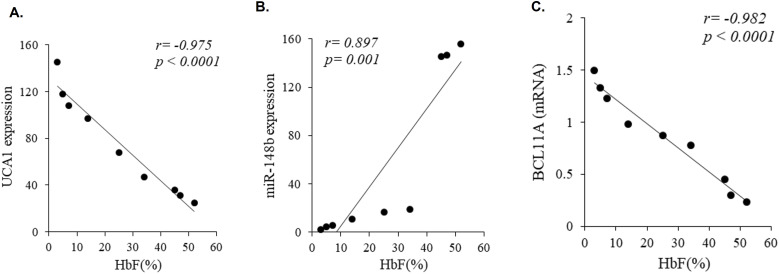
Figure shows the associations between UCA1-lncRNA/miR-148b/BCL11A and HbF levels in independent HbE/β-thalassemia patient cohort. **(A, B, C)** Pearson’s correlation coefficients were used to evaluate the associations between (A) UCA1, (B) miR-148b, and (C) BCL11A and HbF level. Pearson’s correlation coefficient (r) and *P*-value are shown.

To identify crucial miRNAs associated with fetal hemoglobin (HbF) levels in HbE/β-thalassemia disease, we sought to perform miRNA expression profiling using miScript miRNA PCR Array (MIHS-121Z; QIAGEN, GmbH) as shown in the schematic diagram (Fig 3A). A total of 16 HbE/β-thalassemia patient samples with varying HbF levels were collected and confirmed to have β-globin and HbE mutations by Sanger sequencing (Fig 3B). Once confirmed, patients were categorized into two groups, high HbF and normal HbF, based on their γ-globin expression and HPLC profiles. Comparative analysis showed a significant difference in HbF levels between the two groups, with the high-HbF group showing markedly elevated γ-globin expression relative to the normal-HbF group (Fig 3C). To further investigate the physiological relevance of HbF, we correlated patient HbF levels with disease severity. Pearson’s correlation analysis revealed a significant inverse relationship between HbF levels and disease severity (Fig 3D), suggesting a protective and therapeutic role of elevated HbF in ameliorating the disease. Among all patients, those with high HbF (HHbF; HbF >40%) and normal HbF (NHbF; HbF <10%) exhibiting similar genetic mutations were selected for miRNA expression profiling to minimize variability. The demographic and clinical characteristics of these patients are provided in Supplemental information (Table S2). The miRNA expression profiling identified 48 differentially expressed miRNAs between high- and normal-HbF groups (31 up-regulated and 17 down-regulated; Table S3). Parallel profiling in hydroxyurea (HU)-treated K562 cells identified 45 differentially expressed miRNAs (38 up-regulated and 7 down-regulated; Table S4). Heatmap visualization of differentially expressed miRNAs in CD34^+^-derived primary erythroblasts and HU-treated K562 cells is shown in Fig 3E. To further validate the miRNA array results, few selected differentially expressed miRNAs, including miR-148b, let-7b, miR-320a, were reexamined by RT–qPCR analysis in another patient cohort. The expression patterns were consistent with the array-based profiling results (Fig S3). Next, overlapping miRNAs, including miR-148b, miR-320, and let-7b, were prioritized for further analysis. To understand the lncRNA-miRNA-mediated mechanisms, we next sought to identify interactions between lncRNA and miRNAs predicted from our previous analyses. UCA1 has previously been reported to act as sponges to bind miRNAs and therefore regulate their functions in cancer (28). Therefore, we hypothesized that UCA1 may harbor binding sites for differentially expressed miRNAs identified in our study. In order to construct lncRNA-miRNA networks, we examined miRNA expressions in erythroid cells and found that miR-148b showed an opposite expression pattern relative to UCA1. Subsequent bioinformatics analysis identified putative binding sites for miR-148b within the UCA1 transcript. RT–qPCR analyses confirmed inverse expression patterns in HbE/β-thalassemia patients, with UCA1 down-regulated and miR-148b up-regulated in the high-HbF group compared with the normal-HbF group (Fig 3F). Furthermore, correlation analyses showed that UCA1 and BCL11A expression inversely correlated with HbF levels, whereas miR-148b showed a positive correlation with elevated HbF in HbE/β-thalassemia patients (Fig S4), demonstrating a significant association between the UCA1/miR-148b/BCL11A axis and HbF levels in HbE/β-thalassemia patients. Although these findings are consistent with a potential regulatory relationship, they remain correlative and do not fully establish a direct regulatory relationship. Given that subcellular localization provides insight into lncRNA function, we next performed cellular fractionation to determine the distribution of UCA1 in K562 cells. UCA1 was predominantly localized in the cytoplasm (Fig 3G), suggesting its potential role as a miR-148b sponge.

Table S2. Phenotypic and genotypic features of six unrelated HbE/β-thalassemia patients with normal (NHbF) and high (HHbF) fetal hemoglobin levels.

Table S3. Five most significantly up- and down-regulated miRNAs in patient CD34^+^ HSPCs.

Table S4. Five most significantly up- and down-regulated miRNAs in K562 cells.

### UCA1 directly binds with miR-148b

lncRNAs can function as competing endogenous RNAs (ceRNAs) by sequestering shared microRNAs (miRNAs) through complementary base pairing, thereby regulating gene expression posttranscriptionally (29). To experimentally validate whether UCA1 acts as a molecular sponge for miR-148b, a fragment of UCA1 containing the predicted binding sites of miR-148b was cloned downstream of the firefly luciferase gene (luc2) in the pmiRGLO dual-luciferase reporter vector, which also expresses Renilla luciferase (Rluc) for normalization (Fig 3H). The UCA1-pmiRGLO construct was cotransfected with synthetic miR-148b mimics, and luciferase activity was measured to assess direct interactions. Overexpression of miR-148b resulted in a dose-dependent reduction in firefly luciferase activity, indicating direct binding to the UCA1 reporter construct and subsequent repression (Fig 3I). To confirm the specificity of this interaction, a mutant construct with alterations in the miR-148b seed-matching region was generated. Cotransfection of the mutant construct with miR-148b mimics abolished the repressive effect, showing no significant change in luciferase activity compared with controls. Collectively, these findings demonstrate that UCA1 directly interacts with miR-148b through complementary base pairing, supporting a ceRNA-based regulatory mechanism involving the UCA1-miR-148b axis.

### UCA1 negatively regulates fetal hemoglobin expression by sponging miR-148b

**Figure 4.**
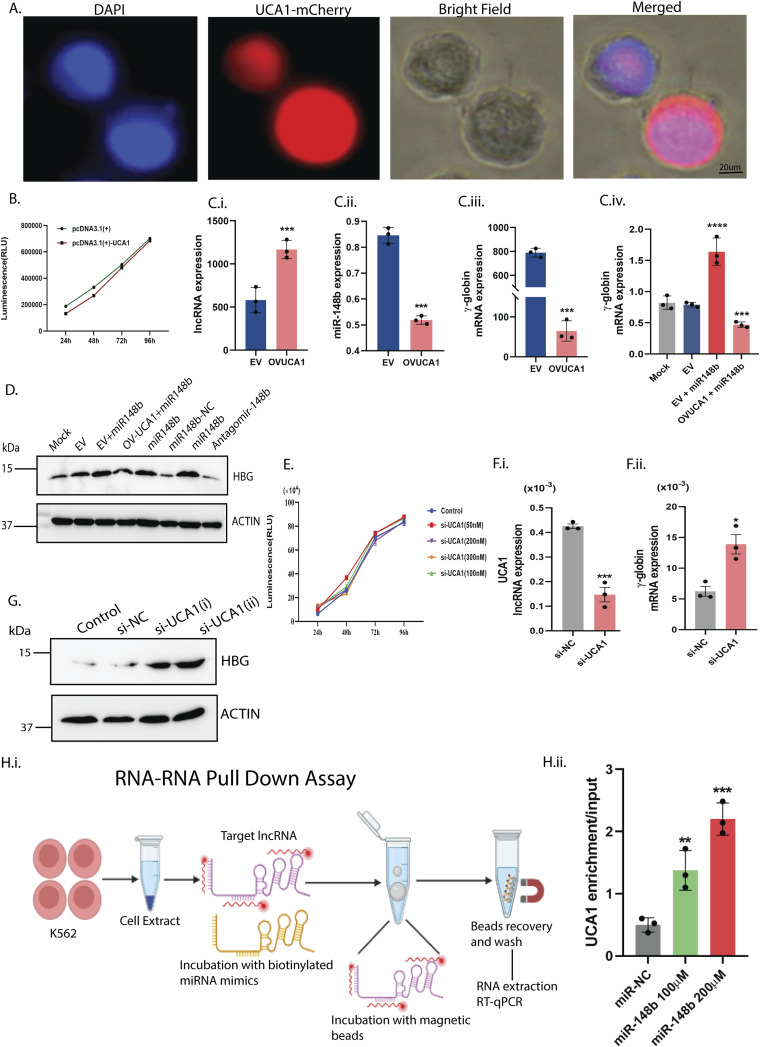
UCA1 overexpression/knockdown modulates γ-globin expression via sequestering miR-148b in K562 cells. **(A)** Representative fluorescence image of K562 cells showing cytoplasmic localization of UCA1-mCherry (red) after UCA1 overexpression, with DAPI staining (blue) marking nuclei (40X magnification). Scale bar, 20 μm. **(B)** (i) Proliferation curves of K562 cells after transfection with EV, pcDNA3.1 (+), or UCA1-pcDNA3.1 (+), as assessed by CellTiter-Glo assay. **(C)** (i, ii, iii) Expression of UCA1, miR-148b, and γ-globin in K562 cells after UCA1 overexpression as assessed by RT–qPCR. (iv) Expression of γ-globin in UCA1-overexpressing K562 cells upon miR-148b mimic transfection as detected by RT–qPCR. β-Actin was as a loading control. **(D)** Representative immunoblot of total protein extracts from K562 cells transfected with scramble miRNA or EV as a control, miR-148b, UCA1 overexpression construct (ovUCA1), ovUCA1 + miR-148b, or antagomir-148b, probed for γ-globin. β-Actin served as a loading control. **(E)** Proliferation curves of K562 cells at different time points post-transfection with varying concentrations (50–300 nM) of siRNA targeting UCA1. **(F)** (i, ii) RT–qPCR analysis showing expression levels of (i) UCA1 and (ii) γ-globin after negative control (si-NC) and si-UCA1 transfection. **(G)** Immunoblot analysis showing expression of γ-globin and β-actin levels in K562 cells post-siRNA transfection at day 3 of differentiation. **(H)** (i) Schematic showing RNA-RNA pull-down assay used to confirm the direct association between UCA1 and miR-148b in K562 cells. (ii) RT–qPCR analysis showing UCA1 enrichment in biotinylated miR-148b compared with scramble (miR-NC) in a dose-dependent manner. β-Actin served as a loading control. Data are representative of n = 3 independent experiments unless otherwise stated, and graphical data are represented as the mean ± SD. *P < 0.05, **P < 0.01, ***P < 0.001, ****P < 0.0001 by an unpaired two-tailed *t* test.

**Figure S5.**
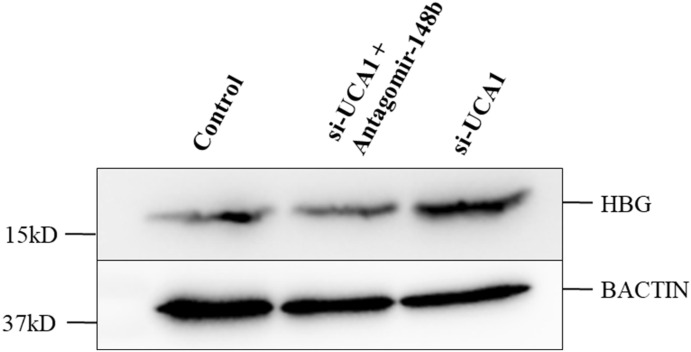
Representative immunoblot of total protein extracts from K562 cells transfected with si-UCA1 or antagomir-148b at day 3 of differentiation, incubated with antibodies against HBG. β-Actin served as the loading control.

To investigate whether UCA1 functions as an endogenous sponge for miR-148b and modulates fetal hemoglobin (HbF) expression, we performed gain- and loss-of-function studies in K562 cells. Full-length UCA1 was transiently overexpressed using a pcDNA3.1-mCherry vector, with successful expression confirmed by fluorescence microscopy (Fig 4A). After transfection, cell viability was assessed, and no significant differences in proliferation were observed compared with control cells (Fig 4B). RT–qPCR analysis confirmed robust overexpression of UCA1, which was associated with a significant reduction in miR-148b and *HBG1/2* expression (hereafter collectively referred to as *HBG* and used interchangeably with γ-globin), indicating that UCA1 suppresses γ-globin expression, likely through sequestration of miR-148b (Fig 4C). Consistently, immunoblot analysis showed reduced γ-globin expression in protein levels in UCA1-overexpressing cells despite cotransfection with miR-148b mimics, recapitulating the effect observed with antagomir-148b cotransfection (Fig 4D). To complement these findings, siRNA-mediated knockdown of UCA1 (si-UCA1) was performed in K562 cells. UCA1 depletion did not significantly affect cell proliferation (Fig 4E), but led to a marked increase in γ-globin expression at both the transcript and protein levels, as determined by RT–qPCR and immunoblot analyses (Fig 4F and G). Notably, this effect was partially rescued by cotransfection with antagomir-148b, which attenuated the induction of HbF after UCA1 silencing, supporting a functional UCA1/miR-148b regulatory axis in γ-globin regulation (Fig S5). To determine whether this regulatory effect is mediated through direct interaction between UCA1 and miR-148b, RNA-RNA pull-down assays were performed using biotinylated miR-148b mimics (Fig 4Hi). The RT–qPCR results show that UCA1 expression was preferentially enriched in the biotinylated miR-148b compared with the nontargeting mimics. These results suggest UCA1 enrichments in biotinylated miR-148b were likely due to the direct association of UCA1 with miR-148b (Fig 4Hii).

### UCA1/miR-148b modulates γ-globin expression without affecting cell cycle progression or apoptosis

**Figure 5.**
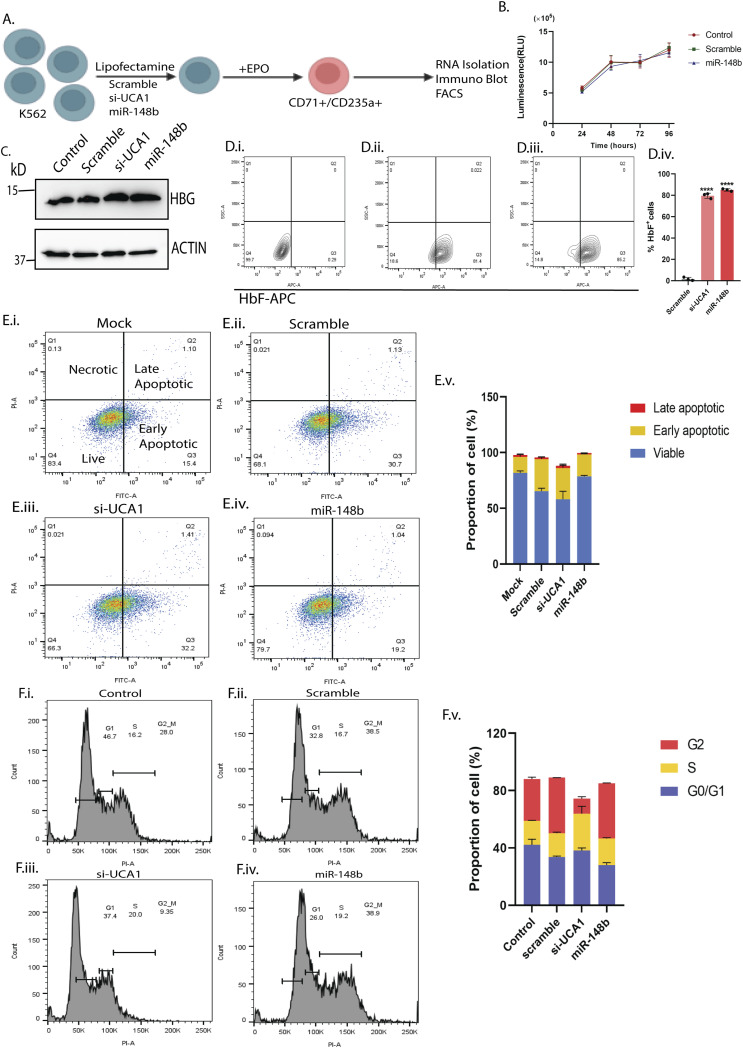
Functional effects of UCA1 knockdown and miR-148b overexpression on apoptosis, cell cycle, and γ-globin induction in K562 cells. **(A)** Schematic overview showing transfections of scramble, miR-148b mimics, and si-UCA1 in differentiated K562 cells (day 3), followed by RNA analysis and in vitro functional assays. **(B)** Proliferation curves of K562 cells after transfection with scramble and miR-148b mimics, as assessed by the CellTiter-Glo assay. **(C)** Representative immunoblot showing γ-globin protein levels in K562 cells transfected with scramble control, si-UCA1, or miR-148b. β-Actin was used as a loading control. **(D)** (i, ii, iii) Quantitative analyses of HbF-positive cells assessed by flow cytometry in K562 cells transfected with (i) scramble control, (ii) si-UCA1, or (iii) miR-148b. (iv) Bar graphs showing the percentage of HbF-positive cells under the indicated conditions. **(E)** (i, ii, iii, iv) Representative FACS plots showing the percentage of apoptotic cells in K562 cells transfected with (i) mock, (ii) scramble control, (iii) si-UCA1, and (iv) miR-148b mimics. (v) Bar graphs showing the proportion of viable K562 cells under the indicated conditions. **(F)** (i, ii, iii, iv) Flow cytometry analysis of cell cycle progression in K562 cells post-transfection. Representative histograms showing the distribution of cells across G1, S, and G2/M phases in (i) mock-, (ii) scramble control–, (iii) si-UCA1–, and (iv) miR-148b mimic–treated cells, respectively. (v) Bar graphs showing the percentage of K562 cells in each phase under the indicated conditions. Data are representative of three independent biological replicates, and graphical data are represented as the mean ± SD. *P < 0.05, **P < 0.01, ***P < 0.001, ****P < 0.0001 by one-way ANOVA.

To directly test whether miR-148b could up-regulate γ-globin expression independently of UCA1, we ectopically overexpressed miR-148b in K562 cells and compared γ-globin expression across three experimental conditions: scramble control, UCA1 knockdown, and miR-148b overexpression as shown in Fig 5A. Similar to si-UCA1, we first evaluated the effects of miR-148b mimics on cell viability and proliferation. No significant differences were observed among cells transfected with miR-148b mimics, nontargeting controls, or untreated cells (Fig 5B), indicating that miR-148b overexpression does not significantly affect cell proliferation. We next sought to examine γ-globin expression after transfection. Immunoblot analysis revealed a significant increase in γ-globin levels in cells transfected with either miR-148b mimics or si-UCA1 compared with nontargeting controls (Fig 5C). This up-regulation was further supported by flow cytometry, which demonstrated a marked increase in the proportion of HbF-positive cells under both conditions (Fig 5D). To assess potential effects on apoptosis and cell cycle progression, we performed flow cytometry analyses post-transfection. The proportions of cells undergoing apoptosis remained nonsignificant in both miR-148b mimic– and si-UCA1–transfected cells compared with controls (Fig 5E), indicating that these perturbations do not induce cell death. Consistently, cell cycle analysis based on DNA content revealed comparable profiles across all conditions, with most of the cells residing in the G2/M phase (Fig 5F), suggesting that neither miR-148b overexpression nor UCA1 knockdown significantly alters cell cycle progression. Collectively, these findings demonstrate that both miR-148b overexpression and UCA1 knockdown can independently elevate γ-globin expression in erythroid cells without inducing apoptosis or perturbing cell cycle progression, highlighting their potential role as therapeutic targets for fetal hemoglobin reactivation.

### BCL11A was confirmed as a direct downstream target of miR-148b through a luciferase reporter assay

**Figure 6.**
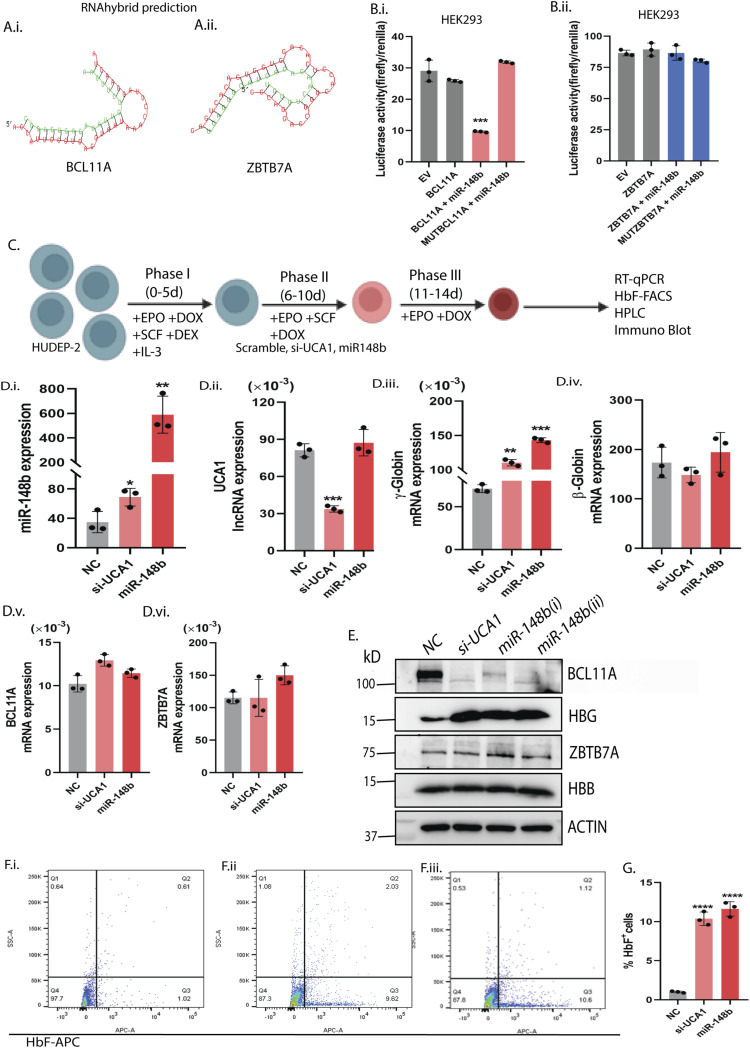
UCA1 knockdown or miR-148b overexpression promotes HbF induction in HUDEP-2 cells through suppression of BCL11A. **(A)** (i, ii) RNAhybrid predictions showing base pairing between miR-148b seed region and 3′ UTR of target genes (i) BCL11A and (ii) ZBTB7A. **(B)** (i) Representative plots showing luciferase assay results, which confirm the direct interactions between miR-148b and BCL11A 3′ UTR (n = 3 independent experiments). (ii) Figure showing luciferase assay results, which confirm no direct interactions between miR-148b and ZBTB7A 3′ UTR (n = 3 independent experiments). **(C)** Experimental workflow showing transfections of scramble, miR-148b mimics, and si-UCA1 in differentiated HUDEP-2 cells, followed by RNA analysis and in vitro functional assays at the indicated time point. **(D)** (i, ii, iii, iv, v, vi) RT–qPCR analysis of (i) miR-148b, (ii) UCA1, (iii) γ-globin, (iv) β-globin, (v) BCL11A, and (vi) ZBTB7A expression in HUDEP-2 cells after miR-148b and si-UCA1 transfections. Results are normalized to endogenous β-actin expression. **(E)** Representative immunoblot of total protein extracts from HUDEP-2 cells transfected with si-UCA1 or miR-148b mimics at day 12 of differentiation, probed for BCL11A, HBG, ZBTB7A, and HBB. β-Actin served as a loading control. **(F)** (i, ii, iii) Representative flow cytometry plots showing HbF staining in HUDEP-2 cells transfected with (i) negative control, (ii) si-UCA1, or (iii) miR-148b at day 12 of differentiation. **(G)** Bar plots showing the percentage of HbF-positive cells quantified by flow cytometry under the indicated conditions. Data are representative of three independent biological replicates, and graphical data are represented as the mean ± SD. *P < 0.05, **P < 0.01, ***P < 0.001, ****P < 0.0001 by one-way ANOVA.

Having identified miR-148b involved in γ-globin regulation, we sought to understand the underlying mechanisms. We next employed the multiMiR R package and database, a comprehensive repository integrating predicted and validated miRNA–target interactions from 14 different sources. This analysis identified a set of candidate genes containing putative miR-148b binding sites within their 3′ untranslated regions (3′ UTRs). Given that miR-148b expression is elevated under high-HbF conditions, we hypothesized that its target genes would exhibit stage-specific expression patterns inversely correlated with miR-148b. To test this, we intersected the predicted target list with genes identified as differentially expressed in our transcriptomic analysis comparing high-HbF and low-HbF conditions. This integrative approach prioritized a subset of genes, including *BCL11A*, *ZBTB7A*, *SOX6*, *TAL1*, and *MYB*, all of which were significantly deregulated under high-HbF conditions and harbored putative miR-148b binding sites within their 3′ UTRs. Among these, BCL11A and ZBTB7A are well-established transcriptional repressors of γ-globin, known to directly bind to the γ-globin promoter. The inverse correlation between miR-148b expression and the expression of these target genes suggests that miR-148b may contribute to γ-globin induction by repressing key transcriptional repressors of HbF. Furthermore, RNAhybrid analysis predicted putative binding interactions between miR-148b and the 3′ UTRs of BCL11A and ZBTB7A, suggesting a potential direct regulatory relationship (Fig 6A). Therefore, these two candidates were prioritized for further experimental validation. To assess whether miR-148b regulates BCL11A and ZBTB7A expression via direct interaction with their 3′ UTRs, we performed luciferase reporter assays. Fragments of the 3′ UTRs containing the predicted miR-148b seed-matching regions were cloned downstream of the firefly luciferase (luc2) gene in the pmiRGLO vector, and cotransfected with either miR-148b mimics or nontargeting control mimics in HEK293 cells. As shown in Fig 6Bi, cotransfection with miR-148b significantly suppressed luciferase activity in the BCL11A-3′ UTR reporter construct compared with the scramble control, indicating direct interactions. Furthermore, deletion of the miR-148b seed site within the BCL11A 3′ UTR abrogated this repression, confirming the specificity of the interaction. Subsequently, luciferase assay using the ZBTB7A-3′ UTR reporter construct showed no such effects in luciferase activity upon miR-148b mimic transfection (Fig 6Bii), suggesting that despite the predicted binding site, ZBTB7A may not be the direct target of miR-148b.

Collectively, these data demonstrate that BCL11A is a bona fide target of miR-148b, which can directly suppress its expression through interaction with the 3′ UTR, thereby potentially contributing to γ-globin derepression. No such regulatory effect was observed for ZBTB7A, highlighting BCL11A-specific role of miR-148b–mediated HbF reactivation.

### UCA1 depletion/miR-148b overexpression elevates fetal hemoglobin expression via BCL11A repression in HUDEP-2 cells

**Figure S6.**
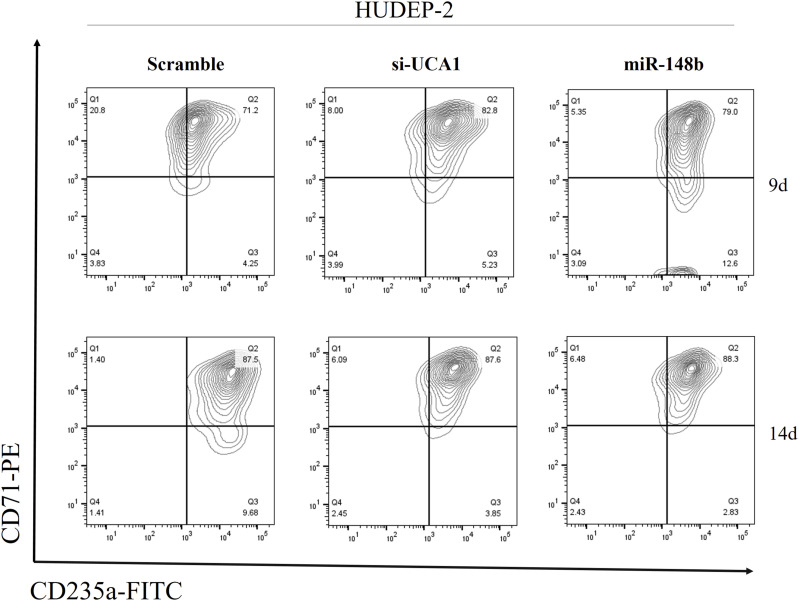
Representative flow cytometry plots showing erythroid maturation of scramble-, si-UCA1–, and miR-148b–transfected HUDEP-2 cells, assessed by CD71^+^ and CD235a+ expression at the indicated time during differentiation.

**Figure S7.**
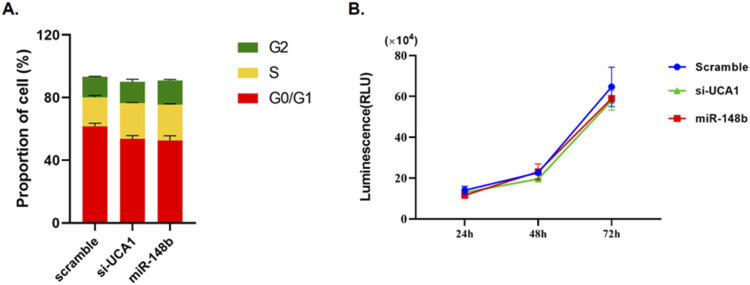
Assessment of cell cycle progression and cellular proliferation following UCA1 knockdown or miR-148b overexpression in HUDEP-2 cells. **(A)** Cell cycle assay of scramble control, si-UCA1, and miR-148b in HUDEP-2 cells post-transfection (day 10). **(B)** Proliferation assay of a scramble control, si-UCA1, and miR-148b in HUDEP-2 cells post-transfection (day 10) using the CellTiter-Glo Luminescent Cell Viability Assay method.

To address the limitations of K562 cells as the adult-stage erythroid model (30), we next sought to validate our findings in adult erythroid cells such as HUDEP-2 and CD34+-derived primary erythroblasts where γ-globin is largely silenced and can be reactivated under defined conditions. To demonstrate that miR-148b directly represses BCL11A expression, we ectopically overexpressed miR-148b in adult-type erythroid HUDEP-2 cells (Fig 6C), with successful overexpression confirmed by RT–qPCR analysis (Fig 6Di). Given our previous finding that the lncRNA UCA1 acts as a molecular sponge for miR-148b, we next examined whether UCA1 knockdown (si-UCA1) could recapitulate the effects of miR-148b overexpression in HUDEP-2 cells, with efficient silencing of UCA1 independently validated by RT–qPCR (Fig 6Dii). Transfection with miR-148b/si-UCA1, but not a nontargeting control, significantly up-regulated γ-globin expression in HUDEP-2 cells (Fig 6Diii). In both conditions, no significant changes were observed in the expression levels of β-globin, BCL11A, or ZBTB7A (Fig 6Div–Vi). Subsequent immunoblot analysis further supported these findings, demonstrating independent HbF induction after both miR-148b overexpression and UCA1 knockdown (Fig 6E). Interestingly, no significant changes were observed in BCL11A transcript levels under both conditions, but BCL11A protein levels were significantly reduced, suggesting posttranscriptional regulation of the BCL11A by UCA1/miR-148b axis. Given that perturbations of the UCA1/miR-148b/BCL11A axis are associated with γ-globin reactivation, we next sought to assess the HbF levels by intracellular flow cytometry analysis. Both miR-148b overexpression and UCA1 knockdown resulted in a significant increase in HbF-positive cells, as determined by flow cytometry (Fig 6F and G). Importantly, erythroid differentiation analysis showed no overt changes upon miR-148b overexpression or UCA1 knockdown, as assessed by flow cytometry (Fig S6). Consistently, cell cycle analysis revealed no significant differences compared with nontargeting controls, indicating no significant effect on cell cycle progression. Furthermore, cell viability assessed by the CellTiter-Glo assay on day 10 of differentiation also remained unchanged (Fig S7). Together, these findings suggest that both miR-148b overexpression and UCA1 knockdown independently elevate γ-globin expression by down-regulating BCL11A expression at the posttranscriptional level, without hampering erythroid development. These results highlight a convergent regulatory axis in which UCA1 functions upstream of miR-148b to modulate fetal hemoglobin expression in adult erythroid cells.

### UCA1 depletion/miR-148b overexpression reactivates fetal hemoglobin in adult primary human erythroblasts

**Figure 7.**
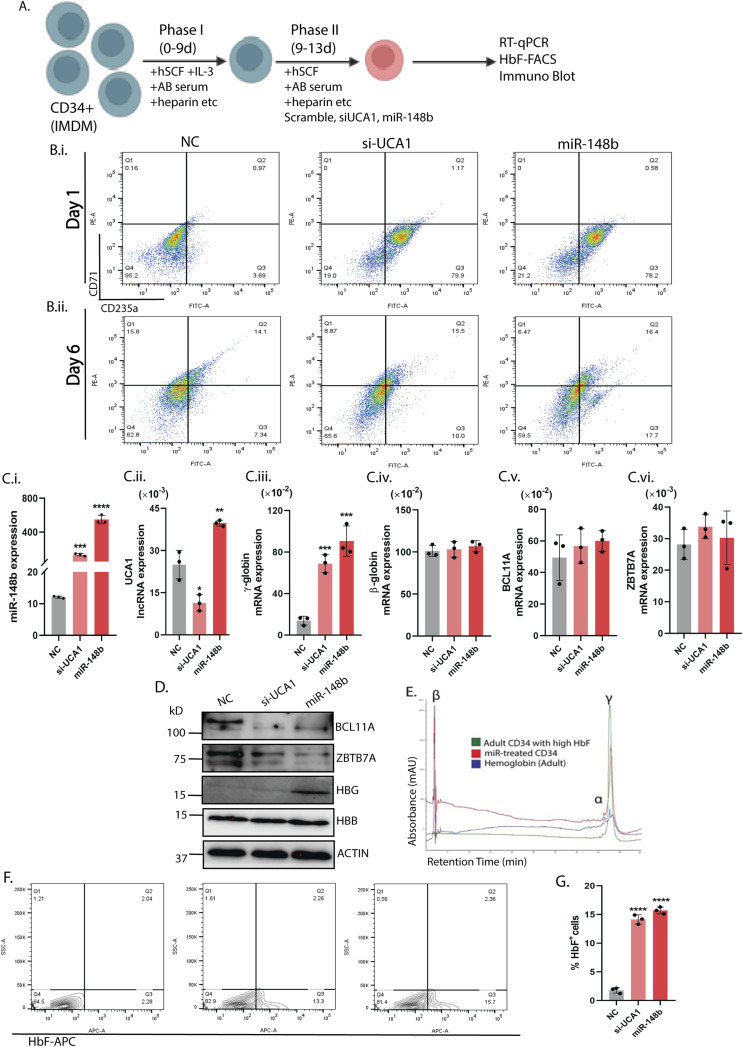
UCA1 knockdown or miR-148b overexpression promotes HbF induction in adult primary erythroblasts. **(A)** Schematic diagram showing in vitro expansion and differentiation of CD34^+^ HSPCs derived from healthy donors, followed by transfection and functional assays at the indicated time point. **(B)** (i, ii) Representative flow cytometry of CD235a and CD71 double staining of primary erythroblasts at day 1 and day 6 of differentiation. **(C)** (i, ii, iii, iv, v, vi) RT–qPCR analysis of (i) miR-148b, (ii) UCA1, (iii) γ-globin, (iv) β-globin, (v) BCL11A, and (vi) ZBTB7A expression in adult human primary erythroblasts after miR-148b and si-UCA1 transfections. Results are normalized to endogenous control and β-actin expression, and shown as the mean ± SD. **(D)** Representative immunoblot of total protein extracts from primary human erythroblasts transfected with scramble control, si-UCA1, or miR-148b mimics at day 13 of differentiation, incubated with the indicated antibodies. β-Actin was used as a protein loading control. **(E)** HbF induction in primary human erythroblasts after miR-148b transfection at day 13 of differentiation, measured by RP-HPLC. Representative RP-HPLC traces showing peaks corresponding to β-globin (β), α-globin (α), and γ-globin (γ). **(F)** (i) Representative flow cytometry plots showing anti-HbF staining in (i) nontargeting control–, (ii) si-UCA1–, and (iii) miR-148b–transfected primary erythroblasts derived from CD34^+^ HSPCs. **(G)** Bar plots showing percentage of HbF-positive cells measured by flow cytometry analysis in (i) nontargeting control–, (ii) si-UCA1–, and (iii) miR-148b–transfected primary erythroblasts derived from CD34^+^ HSPCs. Data are representative of three independent biological replicates, and graphical data are represented as the mean ± SD. *P < 0.05, **P < 0.01, ***P < 0.001, ****P < 0.0001 by one-way ANOVA.

**Figure S8.**
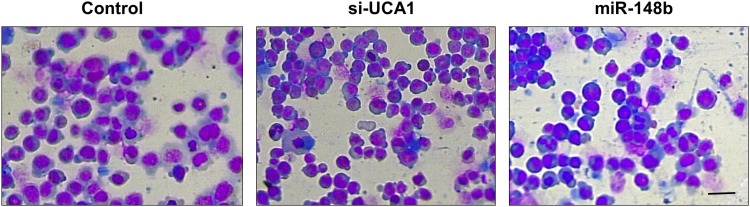
Representative images of May–Grünwald–Giemsa-stained cytospin of CD34+-derived primary erythroblasts from healthy donors: scramble control (left), si-UCA1 (middle), and miR-148b (right) at day 10 of differentiation. Scale bars, 50 μm.

To further validate our findings in a physiologically relevant system, we extended our mechanistic studies to primary human erythroblasts differentiated ex vivo from CD34^+^ HSPCs isolated from healthy adult donors. These primary cells provide a robust model for studying erythroid biology and hemoglobin switching in a setting that closely resembles in vivo erythropoiesis. CD34^+^ HSPCs were cultured under standard erythroid differentiation conditions and transfected with a nontargeting control, synthetic miR-148b mimics, or si-UCA1 (Fig 7A). Cell viability and erythroid maturation were assessed by flow cytometry analysis of CD71 and CD235a expression, together with MGG staining. Notably, neither miR-148b overexpression nor UCA1 knockdown had any negative effect on viability or erythroid differentiation, indicating that modulation of this axis does not impede normal erythropoiesis (Figs 7B and S8). We next examined the impact of these perturbations on γ-globin expression. RT–qPCR analysis revealed a substantial up-regulation of γ-globin after miR-148b overexpression and si-UCA1 treatment compared with controls (Fig 7C). Consistently, immunoblot analysis confirmed increased γ-globin protein levels under both conditions (Fig 7D). In contrast, no significant changes were observed in HBB (β-globin) or ZBTB7A (another known HbF repressor) expression, indicating that miR-148b/UCA1 perturbation selectively affects γ-globin expression without broadly altering the expression of other globin genes or transcriptional repressors. To elucidate the mechanistic basis of γ-globin reactivation, we assessed the impact of these interventions on BCL11A, a master repressor of γ-globin. Consistent with our earlier findings in HUDEP-2 cells, BCL11A protein levels were markedly reduced in miR-148b– and si-UCA1–transfected healthy donor CD34^+^ HSPCs, whereas levels of the BCL11A primary transcript remained unaltered, as determined by RT–qPCR, suggesting posttranscriptional regulation. To assess functional HbF induction, HPLC-based quantification further confirmed elevated γ-globin expression in miR-148b–overexpressing cells, reaching levels comparable to those observed under high-HbF conditions, underscoring its therapeutic potential (Fig 7E). In agreement, flow cytometry analysis using anti-HbF antibody staining demonstrated a significant increase in HbF-positive cells in both miR-148b– and si-UCA1–treated groups compared with controls (Fig 7F and G). Collectively, these findings demonstrate that the miR-148b/UCA1 axis induces γ-globin expression in primary erythroblasts through posttranscriptional repression of BCL11A, leading to derepression of fetal hemoglobin in adult erythroid cells. These results reinforce the role of the miR-148b/UCA1/BCL11A regulatory axis in HbF induction in CD34^+^-derived primary erythroblasts and highlight the therapeutic potential of RNA-based therapeutic strategies for HbF reactivation in β-hemoglobinopathies.

## Discussion

**Figure 8.**
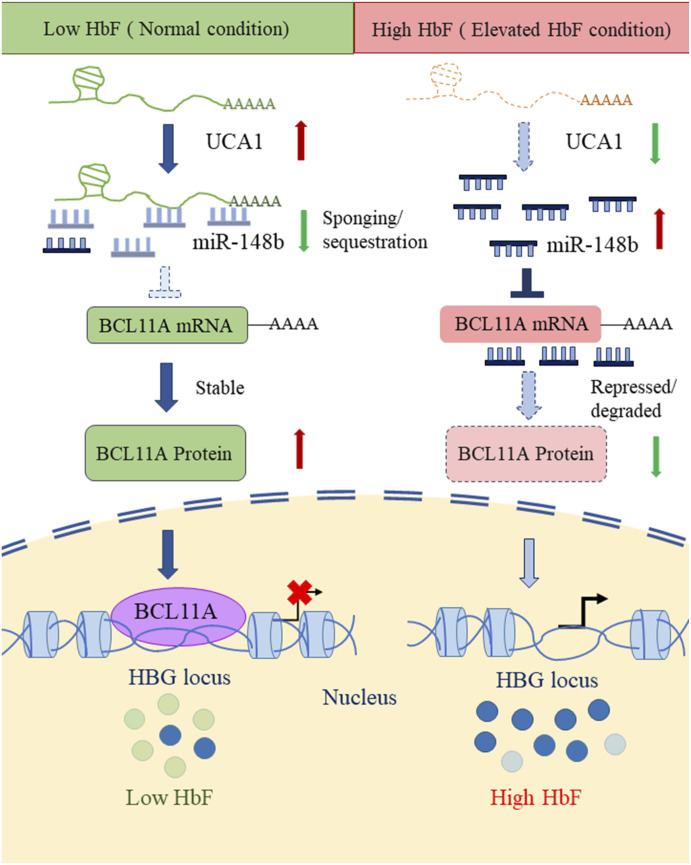
Schematic overview of fetal hemoglobin regulation through the UCA1/miR-148b–mediated competing endogenous RNA (ceRNA) network. In normal (low-HbF) conditions, the lncRNA UCA1 sequesters miR-148b, preventing it from down-regulating BCL11A, a key repressor of HBG (γ-globin). This results in high BCL11A expression and suppression of γ-globin transcription. In contrast, during elevated HbF conditions, UCA1 is down-regulated, leading to increased availability of miR-148b, which represses BCL11A and subsequently derepresses γ-globin genes, thereby promoting fetal hemoglobin induction.

Human fetal hemoglobin (HbF) is developmentally silenced after birth, and reactivation of fetal hemoglobin genes in adult stage could ameliorate severe symptoms associated with major β-hemoglobinopathies, including SCD and β-thalassemia (31). During development, γ-globin genes (*HBG*) are tightly regulated by different cis-regulatory and trans-acting factors, including lineage-specific transcription factors and cofactors such as BCL11A, ZBTB7A, GATA1, KLF1, and MYB (32). Over the past decades, BCL11A has been established as a key repressor of γ-globin and a central regulator of fetal-to-adult hemoglobin switching, with its depletion sufficient to induce γ-globin expression in adult erythroid cells (33). Given its role as a direct regulator of γ-globin expression and its clinical validation as an effective therapeutic target, strategies aimed at modulating its activity or regulation hold substantial promise for mechanism-based therapy (34, 35). However, despite its well-established function in γ-globin silencing, the upstream regulatory networks that govern its expression remain incompletely understood. Here, we identify a posttranscriptional regulatory network involving the lncRNA UCA1 and miR-148b, which modulates BCL11A expression and contributes to the γ-globin silencing in adult erythroid cells (Fig 8). Our study demonstrates that UCA1, a developmentally regulated and abundantly expressed lncRNA in erythroid cells, functions as a molecular sponge for miR-148b. By sequestering miR-148b, UCA1 indirectly elevates BCL11A expression, thereby reinforcing γ-globin silencing. Although prior studies have reported the utility of artificial miRNA sponges and pseudogene-derived decoys in modulating miRNA activity (36, 37), our study shows that UCA1 acts as a naturally occurring lncRNA sponge, orchestrating potent and cell type–specific functions, at least within adult erythroid cells. Given its well-established role in heme biosynthesis through PTBP1 recruitment in erythroid cells (38), our results uncover a previously unstudied function of UCA1 in globin gene switching through modulation of miRNA activity. This context-dependent functional versatility underscores the broader regulatory potential of lncRNAs to coordinate both transcriptional and posttranscriptional gene regulatory mechanisms during development. Although our patient cohort enabled identification of HbF-associated regulatory networks within a clinically comparable HbE/β-thalassemia patient cohort, certain limitations should be considered. Although individuals receiving HbF-inducing therapies were excluded and efforts were made to minimize inclusion of cases with known HbF-modifying conditions based on clinical records, comprehensive genotyping of established HbF-associated loci, including *BCL11A*, *HBS1L-MYB*, and the *HBB* cluster, was not available for this cohort. Given the complex and incompletely resolved genetic architecture underlying HbF variability, the contribution of uncharacterized or unmeasured genetic modifiers cannot be fully excluded. For initial mechanistic exploration, we used K562 cells, which represent an embryonic/fetal-like erythroid state with constitutive γ-globin expression, and therefore do not fully recapitulate adult-stage hemoglobin synthesis (39). To address that, we validated the key findings in adult-stage erythroid models, including human CD34+-derived primary erythroblasts and HUDEP-2 cells. The results are concordant across these erythroid cells, supporting a direct regulatory role of the UCA1/miR-148b axis in modulating γ-globin expression. Another limitation of the study is that although we demonstrate that the exogenous expression of miR-148 reactivates fetal hemoglobin (HbF) in adult erythroid cells by down-regulating BCL11A, the precise mechanism underlying BCL11A repression remains unresolved. Although our findings support a posttranscriptional mode of regulation, the current data do not distinguish whether miR-148 primarily promotes BCL11A mRNA degradation, inhibits its translation, or acts through a combination of both mechanisms. Therefore, the observed reduction in BCL11A expression should not be interpreted as evidence for a specific mode of repression. Future studies employing RNA stability assays, ribosome profiling, or polysome fractionation analyses will be required to delineate the relative contribution of mRNA decay and translational inhibition to miR-148–mediated BCL11A regulation. Notably, the predicted binding sites of miR-148b on UCA1 transcript lack strong evolutionary conservation. Nevertheless, our luciferase assays and cross-linking affinity purification experiments provide supportive evidence of direct interactions, reinforcing the previously established notion that noncanonical and nonconserved miRNA recognition sites can retain functionality in a species- and cell type–specific manner (40, 41). Our results contribute to both the growing list of functional noncanonical sites and provide evidence for the indispensable role of nonconserved seed sites in ncRNA-mediated sponge activity. Moreover, direct evidence of endogenous UCA1/miR-148b interaction through AGO-IP– or CLIP-based assays would further enhance mechanistic confidence, and should be explored in future studies. The limited conservation of these binding sites may constrain direct modeling in nonhuman models such as mice; investigating how this interaction is conserved or diverges across species will be an interesting area to investigate, and may be best addressed using humanized or ex vivo erythroid models.

In summary, this study identifies UCA1 as a previously unrecognized regulator of γ-globin (*HBG*) genes, functioning through miR-148b sequestration to maintain BCL11A expression in adult erythroid cells (Fig 8). Moreover, we found that UCA1 depletion or miR-148b overexpression does not lead to observable changes in either erythroid differentiation or cell proliferation rate of human primary CD34^+^ HSPCs and HUDEP-2 cells. Given that it could selectively down-regulate BCL11A expression in adult erythroid cells, thereby reactivating HbF expression, the UCA1/miR-148b axis might be a promising target to consider for RNA-based therapy for β-hemoglobinopathies. By expanding our understanding of ncRNA-mediated globin gene regulation, this work contributes to the growing appreciation of lncRNAs as critical regulators of globin gene switching, and provides broader implications for developmental gene regulation.

## Materials and Methods

### Study design

Peripheral blood was collected from six unrelated HbE/β-thalassemia patients with varying HbF levels. Primary screening followed by molecular analysis was performed to confirm the disease. All patient samples were categorized into normal-HbF (NHbF: HbF<10%, n = 3) and high-HbF (HHbF: HbF>40%, n = 3) groups. The demographic details and clinical features of the patients were recorded (Table S2). Peripheral blood from another n = 10 unrelated individuals with HbE/β-thalassemia comprising normal- and high-HbF groups was used for correlation analyses. Furthermore, an independent patient cohort consisting of six unrelated HbE/β-thalassemia patients, including NHbF (n = 3) and HHbF (n = 3) groups, was used for RT–qPCR-based validation of miRNA array results. Patients from the primary cohort were assigned clinical scores based on the Mahidol Scoring (42) to assess the association between HbF levels and disease severity. The ethical approval for the study was obtained from IIT Kharagpur and NRS Medical College and Hospital, Kolkata, before sample collection. The approved protocols covered the recruitment and collection of samples from both HbE/β-thalassemia patients and healthy donors (Approval Nos. IIT/SRIC/DR/2019 dated 06.11.2019, IIT/SRIC/DEAN/2022 dated 12.08.2022; No/NMC/3483 dated 12.07.2019; IIT/SRIC/SAO/2017 dated 11.12.2017; No/NMC/4154 dated 03.08.2016; No/NMC/3483, No/NMC/4154, and IIT/SRIC/SAO/2017). The study was conducted according to the guidelines provided by the Declaration of Helsinki, and written informed consent was taken from each participant and/or their legal guardians.

### Cell culture

All cell culture was performed at 37°C with 5% CO_2_ in a water-jacketed CO_2_ incubator under sterile environment. All cell lines were free of *Mycoplasma* contamination.

### K562 culture

Human erythroleukemic cell lines, K562 cells, were cultured in RPMI-1640 medium (Gibco) supplemented with 10% heat-inactivated FBS (VWR), 1% L-glutamine (Gibco), and 1% penicillin–streptomycin (Gibco). K562 cells were induced to differentiate using 3 IU/ml recombinant human erythropoietin (EPO; PeproTech) following the standard protocol (43).

### HUDEP-2 culture

Human umbilical–derived erythroid progenitor-2 (HUDEP-2) cells were maintained in the culture as previously described (44). HUDEP-2 cells were cultured in StemSpan Serum-Free Expansion Medium II (SFEM II; STEMCELL Technologies) supplemented with stem cell factor (SCF; 50 ng/ml; PeproTech), 3 IU/ml hEPO, 1 μM dexamethasone (DEX; Sigma-Aldrich), 1 μg/ml doxycycline (DOX; Sigma-Aldrich), and 2% penicillin–streptomycin. Cells were differentiated in Iscove’s Modified Dulbecco’s Medium (IMDM; Gibco) supplemented with 2% FBS, 3% human blood-type AB serum (Sigma-Aldrich), 3 IU/ml hEPO, 10 µg/ml insulin (I9278; Sigma-Aldrich), 2 IU/ml heparin (Sigma-Aldrich), 500 µg/ml holotransferrin (Sigma-Aldrich), 1% penicillin–streptomycin, 1% L-glutamine, and 1 μg/ml doxycycline. To induce differentiation, HUDEP-2 cells were grown in a three-phase culture condition. Phase I (days 0–5) consisted of early expansion, followed by Phase II (days 6–10) promoting erythroid maturation, and Phase III (days 11–14) supporting terminal differentiation. Media were replenished in every 3–4 d, and cells were harvested at the indicated time points for downstream analyses.

### Ex vivo expansion of primary human erythroblasts

Human peripheral blood samples were obtained from HbE/β-thalassemia patients/healthy donors at Nil Ratan Sircar Medical College and Hospital (NRSMCH), Kolkata. CD34^+^ HSPCs from peripheral blood samples were subsequently isolated using MACS CD34^+^ MicroBead Kit (Miltenyi Biotec) according to the manufacturer’s instructions. For ex vivo expansion, cells were cultured in proliferation media containing SFEM II supplemented with 100 ng/ml SCF, 50 ng/ml FLT3-L (PeproTech), 50 ng/ml thrombopoietin (TPO; PeproTech), 20 ng/ml IL-3(Miltenyi Biotec), and 1% penicillin–streptomycin. To induce proliferation and differentiation, human CD34^+^ HSPCs were cultured in the two-phase culture system. In Phase I, cells were maintained in IMDM supplemented with 100 ng/ml hSCF, 2% penicillin–streptomycin, 1 ng/ml IL-3, 330 μg/ml of holotransferrin, 10 μg/ml of insulin, 5% human AB plasma, 3 IU/ml EPO, and 10 μg/ml of heparin for 9 d. In Phase II, IL-3 was withdrawn and cells were cultured for another 3–4 days.

### HEK293 cell culture

HEK293 cells were cultured in DMEM (Gibco) supplemented with 10% FBS and 1% penicillin–streptomycin–glutamine. Cells were lifted for passaging using 0.05% trypsin–EDTA (Gibco) for 5 min at 37°C.

### Dataset acquisition and processing

In the present study, three independent microarray datasets—GSE13284, GSE71935, and GSE7874—were retrieved from the publicly available Gene Expression Omnibus (GEO) database (45, 46, 47). These datasets comprise samples with high fetal hemoglobin (HbF) levels and corresponding control groups. For lncRNA-based classification, datasets were selected based on the following criteria: (i) availability of expression profiles comparing high-HbF and normal-HbF conditions; (ii) sample size greater than three; and (iii) use of the Affymetrix Human Genome U133 Plus 2.0 Array platform. Raw (.CEL) files were downloaded and preprocessed using the Robust Multichip Average method implemented in the Affy package (Bioconductor, R environment), which included background correction, quantile normalization, and log_2_ transformation. This preprocessed probe ID–centric gene expression matrices were subsequently used for lncRNA-based classification.

### lncRNA expression from Affymetrix Human Genome U133 Plus 2.0 Array

We collected lncRNA annotations from two resources: Reference Sequence (RefSeq) database and Ensembl database, to reannotate the array probe sets of Affymetrix HG-U133 Plus 2.0 Array, and then mapped the probe sets to the human genome for extracting lncRNA expression as described previously by Liu et al (48). Probes with no mismatch and mapped uniquely to the reference genome were considered for further processing. After removing the pseudogene transcripts or protein-coding transcripts, we obtained at least, 2,248 unique lncRNAs that mapped uniquely to their exons. We further performed the Significance Analysis of Microarrays using the R package to evaluate the differentially expressed lncRNAs between the study groups (high HbF vs. normal HbF). The differentially expressed lncRNAs were imported in Cluster view 3.0 to carry out the hierarchical cluster analysis (49).

### lncRNA-based classification and prediction

Machine learning models were developed to classify high versus normal HbF levels using Python 3.7 (scikit-learn). Three datasets (GSE13284, GSE7874, and GSE71935) were combined and split into training and test sample sets. Differentially expressed lncRNAs identified by Significance Analysis of Microarrays were subjected to Sequential Forward Selection using a support vector machine with a radial basis function kernel and fivefold cross-validation to derive an optimal feature set. This lncRNA signature was used to train Random Forest, SVM with linear kernel, and SVM with RBF kernel classifiers. Model performance was evaluated using receiver operating characteristic curves and the corresponding area under the curve.

### Identification of gene modules and hub lncRNAs using WGCNA

We performed WGCNA packages in R, using standard procedures (50). WGCNA is an approach to use gene expression data to construct co-expression–based weighted correlations, and can be used to measure the correlation between lncRNAs and mRNAs in the datasets. This will help us to identify hub lncRNAs, which can be crucial in fetal hemoglobin regulation. lncRNA expression profiles for co-expression analysis were obtained from the GSE13284 dataset. Subsequently, differential expression analysis (DEA) was performed on the same dataset (GSE13284) using the Limma package in R. Genes with |log2FC| > 2 and adjusted *P*-value (FDR) < 0.05 were considered significantly differentially expressed and were selected for further analysis. These differentially expressed genes, together with the predicted lncRNAs, were used as input for the WGCNA. Briefly, we have calculated Pearson’s correlation matrix for all pairwise lncRNAs and, subsequently, a weighted adjacency matrix using the soft-thresholding parameters calculated by the pickSoftThreshold function. Then, the adjacency matrix has been transformed into a topological overlap matrix (TOM) to represent the connectivity of each lncRNA and genes in the module. Hierarchical analysis dendrogram was also performed to merge the genes with a similar co-expression pattern into modules. The networks were visualized using Cytoscape software (version 3.7.0) (51).

### Function enrichment analysis

To elucidate the specific biological functions of differentially expressed genes correlated with candidate lncRNAs, we carried out gene enrichment analysis using the Database for Annotation, Visualization and Integrated Discovery (DAVID) (52). Enriched pathways were further identified and visualized using the Kyoto Encyclopedia of Genes and Genomes pathway enrichment analysis using the DAVID functional annotation tool.

### Drug treatment

To determine the optimal concentration of hydroxyurea (Sigma-Aldrich) treatment for efficient γ-globin induction in K562 cells, cells (2 × 10^5^) were treated with varying concentrations of the drug (50–300 µM) for 24 h. Fetal hemoglobin induction was assessed by RT–PCR, followed by the flow cytometry. The MTT assay (Sigma-Aldrich) was performed to check the viability and proliferation of the cells post-drug treatment.

### MGG staining

For morphological analysis, cells were harvested and smeared on a glass slide by cytospin centrifugation. Slides were stained with May–Grünwald (S-039; HiMedia Laboratories) solution followed by counterstaining with 1:20 diluted Giemsa (S-011; HiMedia Laboratories) solution. Stained cells were analyzed, and images were taken using a Nikon TE2000 microscope equipped with a digital camera.

### RNA isolation and RT–qPCR

Total RNA was extracted using the miRNeasy mini kit (QIAGEN) according to the manufacturer’s instructions. For mRNA, reverse transcription was performed by High-Capacity cDNA Reverse Transcription Kit (Applied Biosystems). PowerUp SYBR Green Master Mix (Applied Biosystems) was used to perform RT-qPCR. For miRNA expression studies, assays were carried out using the miRCURY LNA universal RT miRNA PCR method (QIAGEN). For mRNAs and lncRNAs, the data were normalized using the endogenous β-actin control. Normalization of miRNA expression was performed using Hs_RNU6-2_11 miScript Primer assay (MS00033740; QIAGEN). All real-time PCRs were performed using QuantStudio 5 Real-Time PCR systems (Applied Biosystems). The reactions were performed in at least three different samples in triplicate. Data analysis was carried out using the ΔΔCT method. Primer sequences for RT–qPCR are enlisted in Supplemental information (Table S5).

Table S5. Sequences of primers used for PCR and RT–qPCR analyses.

### Nucleus–cytoplasm fractionation

K562 cells (2 × 10^7^) were harvested and washed with ice-cold PBS for subcellular fractionation. Cells were resuspended in ice-cold cytoplasmic lysis buffer (0.05% NP-40, 0.5 mM DTT, 1 mM EDTA, Hepes [pH 7.9], 10 mM KCL, and 1.5 mM MgCl_2_) and incubated for 5 min on ice. Cell lysates were then spun at 16,000*g* for 10 min at 4°C, and the supernatant was collected as a cytoplasmic fraction. The pellet containing the nuclei was washed with nucleus wash buffer (0.1% Triton X-100, 1 mM EDTA, 100 U RiboLock RNase inhibitor). The final nuclear pellet was resuspended in nucleus lysis buffer (26% [vol/vol] glycerol, 0.2 mM EDTA, 0.5 mM DTT, Hepes [pH 7.9], 300 mM NaCl, and 1.5 mM MgCl_2_) and incubated for 5 min on ice. The supernatant was collected as a nuclear fraction. RNA was extracted from cellular fractionation using the TRIzol method. RT–qPCR was performed to measure the abundance of UCA1-lncRNA in subcellular fractions. The expressions of MALAT1 and ACTB(β-actin) were used as loading control for nuclear RNA and cytoplasmic RNA, respectively.

### Comprehensive miRNA expression analysis

Total RNA was isolated using miRNeasy Mini Kit (QIAGEN) and was reverse-transcribed using miScript II RT Kit (QIAGEN) according to the manufacturer’s protocol. The miScript miRNA PCR Array (QIAGEN, GmbH) in 96-well formats was used for real-time PCR. For expression profiling, 2× QuantiTect SYBR Green PCR Master Mix, 10× miScript Universal Primer, RNase-free water, and template cDNA were mixed according to the manufacturer’s protocol. Cycling conditions for real-time PCR were as follows: initial activation step at 95°C for 15 min, followed by 40 cycles of three-step cycling (denaturation at 94°C for 15 s, annealing at 55°C for 30 s, and extension at 70°C for 30 s). To verify specificity and identity, dissociation curve analysis was performed. RT–qPCR data were normalized using RNU6*-*2. For quality check, PCR array reproducibility and RT efficiency tests were performed using GeneGlobe.

### Computational miRNA target prediction

miRNA sequences, annotations, and distributions were obtained from the miRBase database (53). The target sites of the miRNA were identified using the multiMiR R package and database (54), a comprehensive collection of predicted and validated miRNA target prediction database, which compiled data from 14 different databases. lncRNA-miRNA sponge interaction networks were predicted using the miRspongeR package (55). All interactions were visualized using RNAhybrid (56).

### Cell transfection

Erythroid cells were transfected using Lipofectamine 2000 reagent (Thermo Fisher Scientific) according to the manufacturer’s instructions. The transfections included miR-148b mimic, anti-miR-148b, and a scramble control (each at a final concentration of 100 nM), as well as siRNA targeting UCA1 (final concentration 50 nM). The DsiRNA sequences targeting UCA1 were selected from prevalidated and commercially available siRNA sequences provided by Integrated DNA Technologies (IDT) and synthesized by Eurofins Genomics Services. Cells were seeded in 12-well plates and transfected with either miR-148b mimic, anti-miR-148b (antagomir), scramble control (QIAGEN), or si-UCA1. After 48 h, cells were harvested for immunoblotting and RT–qPCR analyses. All experiments were independently repeated at least three times for statistical validation. The sequences of siRNAs are enlisted in the Supplemental information (Table S6). The siRNA sequence specificity was evaluated using NCBI BLAST against the human RefSeq mRNA database, confirming complete complementarity to the target transcript and no significant homology to other genes.

Table S6. List of oligonucleotide sequences used for cloning and knockdown experiments.

### CellTiter-Glo Cell Viability Assay

To check the cell viability after transfection, cells were harvested from a 12-well plate and seeded in a 96-well opaque culture plate (Corning). CellTiter-Glo reagent (Promega) was added to each well, and the luminescence signal was read after 15 min with GloMax Navigator Microplate Luminometer (Promega).

### Stable transfection of UCA1-lncRNA

The full-length cDNA (1.4 kb) of UCA1 was amplified. The PCR product was digested with BamHI and EcoRI restriction enzymes and subcloned into pcDNA3.1-mCherry (a gift from Prof. David Bartel, plasmid #128744; Addgene) (57) and confirmed by sequencing. Thereafter, the pcDNA3.1-mCherry/UCA1 construct was transfected into K562 cells using Lipofectamine 2000 (Invitrogen) and screened with 1,000 µg/ml geneticin (G418 disulfide; Sigma-Aldrich) for over 7 d. Transfection with pcDNA3.1-mCherry EV (empty vector) acted as a control. The positive clones were confirmed by RT–qPCR for UCA1-lncRNA expression. The primers are enlisted in Table S5.

### Immunofluorescence

For immunofluorescence study, K562 cells were collected in 1X PBS and spun on slides using CS II centrifuge (SLEE). Cells were washed with ice-cold PBS and fixed using 0.05% glutaraldehyde (Sigma-Aldrich) for 10 min. After washing with 1× PBS twice, cells were permeabilized with 0.1% Triton X for 5 min at room temperature. Cells were incubated with specific primary antibody, Hemoglobin γ (D4K7X) Rabbit mAb (1:500 dilution; Cell Signaling Technology), overnight at 4°C followed by Alexa Fluor 594–conjugated secondary antibody (1:2,000, ab150080; Abcam) in the dark for 1 h at room temperature. The nuclear marker DAPI was added for 3–5 min. Fluorescence images were acquired using a Leica Stellaris 5 DMi8 microscope (Leica Microsystems) equipped with a 20× objective lens. Fluorescence images of UCA1-mCherry–transfected K562 cells were acquired using the same microscope with a 40× objective lens. DAPI and Alexa Fluor 594, and mCherry fluorescence signals were acquired using the appropriate excitation and emission settings. Images were processed and analyzed using ImageJ software.

### Dual-luciferase reporter assay

To construct the luciferase reporter vectors, BCL11A and ZBTB7A 3′ untranslated region (UTR) fragments and the sequence of UCA1-lncRNA containing the binding sites for miR-148b were inserted into pmiRGLO Dual-Luciferase miRNA Target Expression Vector (Promega) downstream of the firefly luciferase gene. The target-site mutant vectors for each target were generated by replacing the target sequence with the scramble sequence. The fidelity of all constructs was confirmed by Sanger sequencing before transfection. The WT and mutant reporter vectors were transfected into HEK293 cells, along with miRCURY LNA miRNA Mimics, miR-148b mimics (QIAGEN), using Lipofectamine 2000 Transfection Reagent. The expression of the Renilla luciferase gene was used as an internal reference for transfection efficiency. Cells were lysed 48 h after transfection, and luciferase activity was analyzed using the dual-luciferase reporter assay system (Promega) according to the manufacturer’s instructions. All the experiments were independently repeated at least three times for the statistical validation.

### Flow cytometry

To assess erythroid differentiation, cells were washed twice with phosphate-buffered saline/0.5% BSA and fixed using 0.05% glutaraldehyde. Cells were incubated with PE-conjugated anti-CD71 and FITC-conjugated anti-CD235a antibodies (Miltenyi Biotec) on ice for 30 min in the dark. For HbF staining, cells were fixed with 0.05% glutaraldehyde for 10 min at room temperature and then permeabilized with 0.1% Triton X-100 for 5 min. Cells were stained with APC-conjugated HbF antibody (Miltenyi Biotec) on ice for 20–30 min in the dark. All data were acquired in a BD FACSCanto machine and analyzed using FlowJo v10.6.1 (FlowJo LLC).

### Apoptosis and cell cycle analysis

Transfected cells were harvested after transfection and double-stained with fluorescein isothiocyanate (FITC)–annexin V and propidium iodide (PI) using FITC Annexin V/Dead Cell Apoptosis Kit (Invitrogen) according to the manufacturer’s instructions. Cells were discriminated into viable cells, dead cells, early apoptotic cells, and late apoptotic cells. The percentage of different cells were counted and compared. For cell cycle analysis, cells were stained with propidium iodide (PI) and analyzed by flow cytometry. The percentage of the cells in G0-G1, S, and G2/M phases were calculated and compared. All analyses were performed using FlowJo v10.6.1.

### Immunoblotting

Cells were collected and lysed with RIPA buffer (50 mM Tris–HCl, pH 7.4, 150 mM NaCl, 1% Triton X-100, 0.5% sodium deoxycholate, 0.1% SDS, 1 mM EDTA) following the standard protocol. Equivalent amounts of protein (10–20 µg) were run on the 12% SDS–polyacrylamide gel and transferred to nitrocellulose membrane. Immunoblotting of the membranes was performed using the following primary antibodies: 1:1,000; anti-gamma globin (D4K7X; Cell Signaling Technology), 1:1,000; anti-BCL11A (A5445; ABclonal), 1:1,000; anti-β-Actin (ab8227; Abcam). Signals were evaluated after incubation with appropriate secondary antibody coupled with HRP. Scanned images were quantified using ImageJ software.

### Hemoglobin analysis

The globin chain analysis of erythroid cells was performed as previously described (58). Cells were washed twice with PBS and lysed using 100 μl HPLC-grade water (SRL Chemicals). Cell debris was cleared by centrifugation at maximum speed on a desktop centrifuge. The supernatant (90 μl) was taken and mixed with 10 μl Tris(2-carboxyethyl)phosphine hydrochloride (TCEP.HCl) to break the disulfide bonds. After 5-min incubation at room temperature, 85 μl of 0.1% TFA/32% acetonitrile was added and vortexed briefly. A 50 μl aliquot of the solution was analyzed at 0.7 ml/min flow rate for 50 min using Agilent 1260 Infinity II LC System. Commercially available standard hemoglobins containing HbA, HbD, and HbF were used as reference isotypes. The globin chains were detected at 215 nm.

### RNA-RNA pull-down assay

The UCA1-overexpressing K562 cells were transfected with 100 pmol of biotinylated miR-148b mimics or nonspecific control using Lipofectamine 2000 (Thermo Fisher Scientific) according to the manufacturer’s instructions. Cells were cross-linked with 1% paraformaldehyde in PBS for 10 min at RT under gentle agitation. Cross-linking was quenched by the addition of glycine to a final concentration of 0.125 M, followed by two washes with ice-cold PBS. Cells were collected in PBS, pelleted at 510*g* for 5 min at 4°C, and lysed in lysis buffer containing 50 mM Tris–HCl (pH 7.0), 10 mM EDTA, 1% SDS, RNase inhibitor (200 U/ml), and protease inhibitors (5 μl/ml). Lysates were incubated with streptavidin magnetic beads overnight at RT under moderate agitation to isolate RNA complexes associated with biotinylated miRNAs. Beads were washed five times with wash buffer containing 0.5% SDS in 2× SSC at RT, each with 5-min agitation. Bound complexes were digested with proteinase K (1 mg/ml) in buffer (10 mM Tris–HCl, 100 mM NaCl, 1 mM EDTA, 0.5% SDS) for 45 min at 50°C, followed by 10 min at 95°C to reverse cross-links. RNA was purified using a commercial RNA purification kit including on-column DNase digestion, and RT–qPCR was performed using UCA1-specific primers.

### Statistical analysis

All results were represented as the mean ± SD. Individual data points representing biological replicates were included in the graph. All experiments were repeated at least three times. The comparison between two groups was performed by an unpaired two-tailed *t* test. Differences among more than two groups were evaluated using one-way ANOVA, followed by Bonferroni post hoc analyses as appropriate. All analyses were carried out using GraphPad software 8.0. *P* < 0.05 was considered statistically significant.

## Supplementary Material

Reviewer comments

## Data Availability

The scripts used for differential lncRNA expression analysis, machine learning–based classification, WGCNA, and miRNA target prediction are publicly available at GitHub: https://github.com/RegMedLab/DALMNPred. The patient data are not publicly available because of privacy or ethical restrictions. For original data, please contact nishant@smst.iitkgp.ac.in.
