# Supplementary material for: UCA1 lncRNA regulates γ-globin expression by modulating the miR-148b/BCL11A axis
Source: Life Sci Alliance. 2026 Jun 29;9(9):e202603620. doi: 10.26508/lsa.202603620 (PMC13315483; doi:10.26508/lsa.202603620)
Supplement: Supplementary file 2 [file LSA-2026-03620_TableS1.docx]

| **Significant Positive** |  |  |  |  |
| --- | --- | --- | --- | --- |
| **Row** | **Gene ID** | **Gene Name** | **Score(d)** |  |
| 95 | 1554715_at | DRAIC | 2.0437013764306 | |
| 1162 | 222090_at | NDUFB2-AS1 | 1.68538370889437 | |
| 1789 | 240115_at | PPM1F-AS1 | 1.67859542171533 | |
| 28 | 1553086_at | C11orf40 | 1.63136538695405 | |
| 20 | 1552954_at | LINC02899 | 1.62937369394437 | |
| 716 | 1562597_at | LINC02150 | 1.58376206957499 | |
| 1908 | 242987_x_at | LAMA5-AS1 | 1.55079093882051 | |
| 1103 | 216596_at | LINC02249 | 1.54514911414644 | |
| 1101 | 216473_x_at | DBET | 1.5138023689809 | |
| 81 | 1553935_at | BFSP2-AS1 | 1.48864525059644 | |
| 708 | 1562478_at | LINC00659 | 1.47046939273058 | |
| 1537 | 234293_x_at | RAB4A-AS1 | 1.46118196197715 | |
| 1349 | 230432_at | LINC02532 | 1.4518654546811 | |
| 1875 | 241743_at | ZBTB47-AS1 | 1.41845679914795 | |
| 1508 | 233238_s_at | LINC01933 | 1.41715312017631 | |
| 514 | 1560550_at | LINC01644 | 1.4165684639935 | |
| 1369 | 230743_at | HOXB-AS3 | 1.39012580215454 | |
| 134 | 1555994_at | DIAPH3-AS1 | 1.38923460999856 | |
| 1492 | 232897_at | LERFS | 1.38535652775818 | |
| 1472 | 232571_at | CAPN10-DT | 1.38186863924982 | |
|  |  |  |  |  |
| **Significant Negative** |  |  |  |  |
| **Row** | **Gene ID** | **Gene Name** | **Score(d)** |  |
| 1381 | 230944_at | C6orf223 | -2.38780262879588 | |
| 1246 | 227925_at | GSEC | -1.79483433782607 | |
| 1245 | 227919_at | UCA1 | -1.71932097321119 | |
| 160 | 1556406_at | LINC00879 | -1.71603145040091 | |
| 1201 | 225457_s_at | PP7080 | -1.70201243115296 | |
| 1741 | 239113_at | CT66 | -1.68085518235983 | |
| 1368 | 230710_at | MIR210HG | -1.66092713399978 | |
| 1290 | 229090_at | ZEB1-AS1 | -1.65242417165371 | |
| 1446 | 231954_at | MIR4453HG | -1.64110553679087 | |
| 1699 | 238021_s_at | CRNDE | -1.60334312036694 | |
| 1280 | 228839_s_at | LINC00863 | -1.59751875990652 | |
| 989 | 1569807_at | LINC01741 | -1.54994192087381 | |
| 726 | 1562655_at | SCUBE1-AS1 | -1.50779860571192 | |
| 649 | 1561565_at | LINC02530 | -1.46763779701633 | |
| 1090 | 215590_x_at | ACVR2B-AS1 | -1.44870642262329 | |
| 185 | 1556677_at | ZFY-AS1 | -1.42882118868754 | |
| 1450 | 232239_at | LINC00865 | -1.42237920007859 | |

**Table S1.** List of differentially expressed lncRNAs identified from normalized data using the Significance Analysis of Microarrays (SAM).
