# Supplementary material for: UCA1 lncRNA regulates γ-globin expression by modulating the miR-148b/BCL11A axis
Source: Life Sci Alliance. 2026 Jun 29;9(9):e202603620. doi: 10.26508/lsa.202603620 (PMC13315483; doi:10.26508/lsa.202603620)
Supplement: Supplementary file 3 [file LSA-2026-03620_TableS2.docx]

| **Characteristics** | **HHBF1** | **HHBF2** | **HHBF3** | **NHBF1** | **NHBF2** | **NHBF3** |
| --- | --- | --- | --- | --- | --- | --- |
| Age(years)/sex | 6/F | 15/M | 9/F | 15/F | 6/M | 16/M |
| Age of onset(years) | 2.5 | 2 | 3 | 3 | 2 | 1.5 |
| No. of transfusion/year | 0 | 0 | 0 | 12 | 12 | 8 |
| Hb(g/dL)^a^ | 7.8 | 7.6 | 8.2 | 6.5 | 7.2 | 7.4 |
| HbF (%) | 52.5 | 47.7 | 45.3 | 5.6 | 3.1 | 6.9 |
| HbA0 | 0.5 | 0 | 0 | 26.9 | 32.1 | 12 |
| HbA2+E (%) | 45.7 | 35.5 | 53.2 | 57.2 | 52.5 | 75.2 |
| MCV(fL) | 67 | 65.4 | 66 | 70.2 | 68.9 | 72 |
| RDW | 29.8 | 28.6 | 28.9 | 29.8 | 30.9 | 35.6 |
| MCH(pg) | 20.5 | 20 | 19.8 | 20.8 | 21.4 | 22.5 |
| **HBB genotype*** | IVS-1-5(G>C)/cd26 | IVS-1-5(G>C)/cd26 | IVS-1-5(G>C)/cd26 | IVS-1-5(G>C)/cd26 | IVS-1-5(G>C)/cd26 | IVS-1-5(G>C)/cd26 |

**Table S2.** Phenotypic and genotypic features of six unrelated HbEβ-thalassemia patients with normal (NHbF) and high (HHbF) fetal hemoglobin levels.

M, male; F, female; No., number; Hb, hemoglobin; HbA₂, hemoglobin A₂; HbF, fetal hemoglobin; HbA₀, adult hemoglobin; E, hemoglobin E; MCV, mean corpuscular volume; MCH, mean corpuscular hemoglobin; RDW, red cell distribution width; HBB genotype, β-globin genotype.

^a^ Hematological parameters were collected prior to blood transfusion to accurately reflect the endogenous physiological status of the individuals.

*Genotyping was performed using Sanger sequencing method.
