# Supplementary material for: UCA1 lncRNA regulates γ-globin expression by modulating the miR-148b/BCL11A axis
Source: Life Sci Alliance. 2026 Jun 29;9(9):e202603620. doi: 10.26508/lsa.202603620 (PMC13315483; doi:10.26508/lsa.202603620)
Supplement: Supplementary file 4 [file LSA-2026-03620_TableS3.docx]

| **Mature ID** | **Fold Regulation** | **p-value** | **miScript Catalog** |
| --- | --- | --- | --- |
| **Up-Regulation** |  |  |  |
| hsa-miR-218-5p | 2.1594 | 0.015544 | MIMAT0000275 |
| hsa-miR-148b-3p | 1.8204 | 0.027273 | MIMAT0000759 |
| hsa-miR-146a-5p | 1.2652 | 0.01769 | MIMAT0000449 |
| hsa-miR-26a-5p | 1.0144 | 0.000831 | MIMAT0000082 |
| hsa-miR-125b-5p | 1.0028 | 0.013907 | MIMAT0000423 |
| **Down-Regulation** |  |  |  |
| hsa-miR-320a | -2.03 | 0.042007 | MIMAT0000510 |
| hsa-miR-302c-3p | -1.8311 | 0.022462 | MIMAT0000717 |
| hsa-let-7b-5p | -1.0688 | 0.023121 | MS00003122 |
| hsa-miR-15b-5p | -1.0229 | 0.004408 | MIMAT0000417 |
| hsa-miR-92a-3p | -1.0004 | 0.012087 | MIMAT0000092 |

**Table S3.** The five most significantly up- and down-regulated miRNAs in patient CD34+ HSPCs.
