# Supplementary material for: UCA1 lncRNA regulates γ-globin expression by modulating the miR-148b/BCL11A axis
Source: Life Sci Alliance. 2026 Jun 29;9(9):e202603620. doi: 10.26508/lsa.202603620 (PMC13315483; doi:10.26508/lsa.202603620)
Supplement: Supplementary file 5 [file LSA-2026-03620_TableS4.docx]

| **Mature ID** | **Fold Regulation** | **p-value** | **miScript Catalog** |
| --- | --- | --- | --- |
| **Up-Regulation** |  |  |  |
| hsa-miR-98-5p | 4.6 | 0.00087 | MIMAT0000096 |
| hsa-miR-375 | 3.96 | 0.009343594 | MS00004088 |
| hsa-miR-148b-3p | 3.39 | 0.0303 | MIMAT0000759 |
| hsa-miR-185-5p | 3.34 | 0.000488049 | MS00003647 |
| hsa-miR-124-3p | 3.3 | 0.002721251 | MS00006622 |
| **Down-Regulation** |  |  |  |
| hsa-miR-92a-3p | -3 | 0.0043 | MIMAT0000092 |
| hsa-let-7b-5p | -3.03 | 0.003802762 | MS00003122 |
| hsa-miR-320a | -3.03 | 0.0087 | MIMAT0000510 |
| hsa-miR-7-5p | -3.11 | 0.000680456 | MS00006503 |
| hsa-miR-142-3p | -3.66 | 0.010521821 | MS00006664 |

**Table S4.** The five most significantly up- and down-regulated miRNAs in K562 cells.
