# Supplementary material for: UCA1 lncRNA regulates γ-globin expression by modulating the miR-148b/BCL11A axis
Source: Life Sci Alliance. 2026 Jun 29;9(9):e202603620. doi: 10.26508/lsa.202603620 (PMC13315483; doi:10.26508/lsa.202603620)
Supplement: Supplementary file 6 [file LSA-2026-03620_TableS5.docx]

**Table S5.** Sequences of primers used for PCR and RT-qPCR analyses.

| **Gene name** | **NCBI Gene Id** | **Primer sequence (5' > 3')** |
| --- | --- | --- |
| **Endogenous control (Housekeeping) genes(RT-qPCR)** | |  |
| Beta-actin(Cytoplasmic Fraction) | X00351.1 | F-ACTGGAACGGTGAAGGTGACA |
|  |  | R-AGTCCTCGGCCACATTGTGAA |
| Malat1(Nuclear Fraction) | NR_002819.5 | F-AGCAAACTGTGTTGGCGTGG |
|  |  | R-CGGTGCCTTTAGTGAGGGGT |
| **Globin genes(RT-qPCR)** |  |  |
| HBG | NM_000184.3 | F-AAGCTCCTAGTCCAGACGCC |
|  |  | R-AGACAACCAGGAGCCTTCCC |
| HBB | NM_000518.4 | F-TGGATGAAGTTGGTGGTGAG |
|  |  | R-CCTTAGGGTTGCCCATAACA |
| HBA1 | NM_000558.5 | F-CCCGGTCAACTTCAAGCTCCTA |
|  |  | R-AAGAAGCATGGCCACCGAGG |
| HBA2 | NM_000517.6 | F-CCGGTCAACTTCAAGCTCCTA |
|  |  | R-AGGAGGAACGGCTACCGAG |
| **miRNA target genes(RT-qPCR)** |  |  |
| BCL11A | NM_001405730.1 | F-GACGCAGCGACACTTGTTCT |
|  |  | R-GCTCTCGAGCTTCCATCCGA |
| ZBTB7A | NM_015898.4 | F-ATCCGAGCCAAGGCCTTCCA |
|  |  | R-ACCTTCAGCTTGTCCTGCCTGG |
| **LncRNA Primers(RT-qPCR)** |  |  |
| ZEB1-AS1 | NR_024284.1 | F-CTACGGCCGGAACCTTGTTG |
|  |  | R-AAACCAGGCGTCCCTTTCCAA |
| UCA1 | NR_015379.3 | F-GCCGAGAGCCGATCAGACAAA |
|  |  | R-GCTGGGATGGCCATTTGGAAG |
| GSEC | NR_033839.1 | F-GCCTGATGGGGATACCTTCC |
|  |  | R-CAAGGCCAGGGTTTAGGTGA |
| MIR4453HG | NR_033797.2 | F-TCCCCCTAGCCATGAAAGGA |
|  |  | R-CTTGCTCGGCATTTCGTCTC |
| BGLT3 | NR_121648.1 | F-CAGGGGTAACACACAAACCAGC |
|  |  | R-CACACTTCCACCGGCAGAGA |
| **Erythroid differentiation markers(RT-qPCR)** | |  |
|  |  |  |
| GATA1 | NM_002049.4 | F-GCCACTACCTATGCAACGCC |
|  |  | R-CCCGTTTACTGACAATCAGGC |
| BAND3 | X12609.1 | F-AACGTAGCTGGTCGCAGAG |
|  |  | R-TGTCTACGGTGATCTGAGCC |
| ALAS2 | NM_000032.5 | F-AGGAAGCCATTTTCCGGTCC |
|  |  | R-ACTGAAGACATAGTTTCCAGGC |
| **Overexpression UCA1(PCR)** | |  |
| OUCA1-F(BamH1) | NR_015379.3 | F-CGGGATCCTGACATTCTTCTGGACAAT |
| OUCA1-R(EcoR1) |  | R-GGAATTCGGCATATTAGCTTTAATGTAGG |
| **Beta-globin sequencing primers(PCR)** | |  |
| BetaSeq-F |  | F-GCATATTCTGGAGACGCAGGAAG |
| BetaSeq-R |  | R-CATCAAGGGTCCCATAGACTCACC |
