# Supplementary material for: UCA1 lncRNA regulates γ-globin expression by modulating the miR-148b/BCL11A axis
Source: Life Sci Alliance. 2026 Jun 29;9(9):e202603620. doi: 10.26508/lsa.202603620 (PMC13315483; doi:10.26508/lsa.202603620)
Supplement: Supplementary file 7 [file LSA-2026-03620_TableS6.docx]

| **Gene Name** | **Oligonucleotide sequence(5'>3')** |
| --- | --- |
| BCL11A-F | CGCGGCCGCAGCACTGGGTGAGGTAATAAACCTTAGGAACTAT |
| BCL11A-R | CTAGATAGTTCCTAAGGTTTATTACCTCACCCAGTGCTGCGGCCGCGAGCT |
| MutBCL11A-F | CGCGGCCGC AGATGGGTGAGGTAATAAACCTTAGGAACTAT |
| MutBCL11A-R | CTAGATAGTTCCTAAGGTTTATTACCTCACCCATCTGCGGCCGCGAGCT |
|  |  |
| ZBTB7A-F | CGCGGCCGCGGCAGGAGCGGTCACCTCACAGGTGGTGACACTGAGT |
| ZBTB7A-R | CTAGACTCAGTGTCACCACCTGTGAGGTGACCGCTCCTGCCGCGGCCGCGAGCT |
| MutZBTB7A-F | CGCGGCCGCGGCAGGAGCGGTCACCTCACAGGTGGTGACAGAGT |
| MutZBTB7A-R | CTAGACTCTGTCACCACCTGTGAGGTGACCGCTCCTGCCGCGGCCGCGAGCT |
|  |  |
| UCA1-F | CCCTCTCCTATCTCCCTTCACTGACTCTCTTTTCGGACTCAT |
| UCA1-R | CTAGATGAGTCCGAAAAGAGAGTCAGTGAAGGGAGATAGGAGAGGGAGCT |
| MutUCA1-F | CCCTCTCCTATCTCCCTTCTTACTCTCTTTTCGGACTCAT |
| MutUCA1-R | CTAGATGAGTCCGAAAAGAGAGTAAGAAGGGAGATAGGAGAGGGAGCT |
|  |  |
| si-UCA1 | CUGCAAUCAGAACUAUUGAACUUCU |
|  | AGAAGUUCAAUAGUUCUGAUUGCAGAU |

**Table S6**. List of oligonucleotide sequences used for cloning and knock-down experiments.
