# Supplementary material for: UCA1 lncRNA regulates γ-globin expression by modulating the miR-148b/BCL11A axis
Source: Life Sci Alliance. 2026 Jun 29;9(9):e202603620. doi: 10.26508/lsa.202603620 (PMC13315483; doi:10.26508/lsa.202603620)
Supplement: Reviewer comments [file LSA-2026-03620_review_history.pdf]

Life Science Alliance

UCA1 lncRNA regulates γ-globin expression by modulating the miR-148b/BCL11A axis

Motiur Rahaman, Shatarupa Bhattacharya, Mandrita Mukherjee, Chiranjib Bhowmick, Praphulla Chandra Shukla, Tuphan Kanti Dolai and Nishant Chakravorty

DOI: https://doi.org/10.26508/lsa.202603620

Corresponding author(s): Dr. Nishant Chakravorty (Indian Institute of Technology Kharagpur)

Review Timeline:

Submission Date:	2026-01-08
Editorial Decision:	2026-02-09
Revision Received:	2026-05-13
Editorial Decision:	2026-06-03
Revision Received:	2026-06-13
Accepted:	2026-06-18

Scientific Editor: Sarita Hebbar

Transaction Report:

(Note: With the exception of the correction of typographical or spelling errors that could be a source of ambiguity, letters and reports are not edited. The original formatting of letters and referee reports may not be reflected in this compilation.)

No full Peer Review Process File is available with this article, as the authors have chosen not to make the review process public in this case.

February 9, 2026

Re: Life Science Alliance manuscript #LSA-2026-03620-T

Dr. Nishant Chakravorty
IIT Kharagpur
School of Medical Science and Technology
SMST, IIT Kharagpur
Kharagpur, West Bengal 721302
INDIA

Dear Dr. Chakravorty,

Thank you for submitting your manuscript entitled "UCA1 lncRNA regulates γ-globin expression by modulating the miR-148b/BCL11A axis" to Life Science Alliance. Your manuscript was assessed by three expert reviewers, whose comments are appended to this letter.

I would be happy to discuss the revision in more detail via email or phone/videoconferencing. Please let me know which option you prefer, if any.

To upload the revised version of your manuscript, please log in to your account: https://lsa.msubmit.net/cgi-bin/main.plex

You will be guided to complete the submission of your revised manuscript and to fill in all necessary information. Please get in touch in case you do not know or remember your login name.

The typical timeframe for revisions is three months. Please note that papers are generally considered through only one revision cycle, so strong support from the referees on the revised version is needed for acceptance.

While you are revising your manuscript, please also attend to the below editorial points to help expedite the publication of your manuscript. Please direct any editorial questions to the journal office. When submitting the revision, please include a letter addressing the reviewers' comments point by point. While a rebuttal must respond to all points in some form, additional experiments to resolve these points, other than indicated above, will not be required.

Thank you for this interesting contribution to Life Science Alliance. We hope that the comments below will prove constructive as your work progresses, and we are looking forward to receiving your revised manuscript.

Sincerely,

Sarita Hebbar, PhD
Scientific Editor
Life Science Alliance
http://www.lsajournal.org

A. THESE ITEMS ARE REQUIRED FOR REVISIONS

-- A letter addressing the reviewers' comments point by point.

-- An editable version of the final text (.DOC or .DOCX) is needed for copyediting (no PDFs).

-- High-resolution figure, supplementary figure and video files uploaded as individual files: See our detailed guidelines

for preparing your production-ready images, https://www.life-science-alliance.org/authors

-- Summary blurb (enter in submission system): A short text summarizing in a single sentence the study (max. 200 characters including spaces). This text is used in conjunction with the titles of papers, hence should be informative and complementary to the title and running title. It should describe the context and significance of the findings for a general readership; it should be written in the present tense and refer to the work in the third person. Author names should not be mentioned.

-- By submitting a revision, you attest that you are aware of our payment policies found here: https://www.life-science-alliance.org/copyright-license-fee

B. MANUSCRIPT ORGANIZATION AND FORMATTING:

Full guidelines are available on our Instructions for Authors page, https://www.life-science-alliance.org/authors

We encourage our authors to provide original source data, particularly uncropped/-processed electrophoretic blots and spreadsheets for the main figures of the manuscript. If you would like to add source data, we would welcome one PDF/Excel-file per figure for this information. These files will be linked as supplementary "Source Data" files.

IMPORTANT: It is Life Science Alliance policy that if requested, original data images must be made available. Failure to provide original images upon request will result in unavoidable delays in publication. Please ensure that you have access to all original microscopy and blot data images before submitting your revision.

June 3, 2026

RE: Life Science Alliance Manuscript #LSA-2026-03620-TR

Dr. Nishant Chakravorty
Indian Institute of Technology Kharagpur
School of Medical Science and Technology
SMST, IIT Kharagpur
Kharagpur, West Bengal 721302
India

Dear Dr. Chakravorty,

Thank you for submitting your revised manuscript entitled "UCA1 lncRNA regulates γ-globin expression by modulating the miR-148b/BCL11A axis".
Your revised manuscript was evaluated by two of the original reviewers, whose comments are included below. In line with the reviewers' overall evaluation, we would be happy to publish your paper in Life Science Alliance pending resolution of the above minor points and final revisions necessary to meet our formatting guidelines.

MANUSCRIPT ORGANIZATION AND FORMATTING:

To avoid unnecessary delays in the acceptance and publication of your paper, please read the following information carefully. Full guidelines are available on our Instructions for Authors page, https://www.life-science-alliance.org/authors

Submission of a paper that does not conform to Life Science Alliance guidelines will delay the acceptance of your manuscript.
-Thank you for providing a statement on data sharing. We request you to change that section heading to 'Data Availability' in alignment with LSA guidelines.
Please extend this section to include information (repository name and accession number) on relevant source code(s) for machine learning models such that readers can understand and replicate your findings if needed.
-Thank you for providing information identifying the committee approving the experiments involving human subjects. We encourage you to move these sentences (lines 856-860) under the section titled, 'Study Design'. We noted that this section is lacking information on healthy donors. We request you to confirm that provided ethics statements also covers healthy human subjects. If you prefer, you could also include this information in the relevant section on expansion of primary human erythroblasts (line 566).
-Kindly label axes in Figure 7E.
-Please include information on primers for reference/house keeping genes in Table S5.
-Please expand the statement, "The cells were observed using fluorescent microscopy", line 750, by including imaging details (microscope, information on objectives, excitation/emission filters).
-Please name the bioinformatics software for implementation of Robust Multichip Average method (line 591).
-Please include a brief description of differentiation of K562 cells or provide a citation for this statement starting at line 549.
-Please upload all figure files as individual ones, including the supplementary figure files; all figure legends should only appear in the main manuscript file.
-Please add the X and Bluesky handles of your host institute/organization, as well as your own, and/or one of the authors, in our system.
-Please rename "COMPETING INTERESTS" to "Conflict of INTERESTS."
-Legend for figure S6 is missing, please add it to the list of the figure legends in the manuscript text.
-Please remove the file with supplementary information. Supplementary figures and Tables upload separately and individually.
-Please add your main, supplementary figure, and table legends to the main manuscript text after the references section.

-There are call-outs for figure S3A-D, and this figure doesn't have panels. Please correct.
-Please be sure that the authorship listing and order is correct.

We encourage our authors to provide original source data, particularly uncropped/-processed electrophoretic blots and spreadsheets for the main figures of the manuscript. If you would like to add source data, we would welcome one PDF/Excel-file per figure for this information. These files will be linked as supplementary "Source Data" files.

It is Life Science Alliance policy that if requested, original data images must be made available to the editors. Failure to provide original images upon request will result in unavoidable delays in publication. Please ensure that you have access to all original data images prior to final submission.

If you are planning a press release on your work, please inform us immediately to allow informing our production team and scheduling a release date.

We welcome submissions of potential cover images for the issue of LSA in which your work would appear. If you have high quality images associated with this work, please feel free to email these, with a caption, to the journal office.

LSA encourages authors to provide a 30-60 second video where the study is briefly explained. These videos will be appear embedded with the manuscript online at Life Science Alliance, and on social media to promote the published paper and authors (for examples, see https://docs.google.com/document/d/1-UWCfbE4pGcDdcgzcmiuJl2XMBJnxKYeqRvLLrLSo8s/edit?usp=sharing). Corresponding or first-authors are welcome to submit the video. Please submit only one video per manuscript. The video can be emailed to contact@life-science-alliance.org

FINAL FILES:

To upload the final version of your manuscript, please log in to your account: https://lsa.msubmit.net/cgi-bin/main.plex
You will be guided to complete the submission of your revised manuscript and to fill in all necessary information.
Please get in touch in case you do not know or remember your login name.

The following items are required for acceptance.

-- An editable version of the final text (.DOC or .DOCX) is needed for copyediting (no PDFs).

-- High-resolution figure, supplementary figure and video files uploaded as individual files: See our detailed guidelines for preparing your production-ready images, https://www.life-science-alliance.org/authors

-- Summary blurb (enter in submission system): A short text summarizing in a single sentence the study (max. 200 characters including spaces). This text is used in conjunction with the titles of papers, hence should be informative and complementary to the title. It should describe the context and significance of the findings for a general readership; it should be written in the present tense and refer to the work in the third person. Author names should not be mentioned.

The license to publish form must be signed before your manuscript can be sent to production. A link to the license to publish form will be available to the corresponding author only. Please take a moment to check your funder requirements.

Reviews, decision letters, and point-by-point responses associated with peer-review at Life Science Alliance will be published, alongside the manuscript. If you do want to opt out of having the reviewer reports and your point-by-point responses displayed, please let us know immediately.

Thank you for your attention to these final processing requirements. Please revise and format the manuscript and upload materials as soon as you are able.

Thank you for this interesting contribution to the literature. We look forward to publishing your paper in Life Science Alliance.

Sincerely,

Sarita Hebbar, PhD
Scientific Editor
Life Science Alliance
http://www.lsajournal.org

June 18, 2026

RE: Life Science Alliance Manuscript #LSA-2026-03620-TRR

Dr. Nishant Chakravorty
Indian Institute of Technology Kharagpur
School of Medical Science and Technology
SMST, IIT Kharagpur
Kharagpur, West Bengal 721302
India

Dear Dr. Chakravorty,

Thank you for submitting your Research Article entitled "UCA1 lncRNA regulates γ-globin expression by modulating the miR-148b/BCL11A axis". It is a pleasure to let you know that your manuscript is now accepted for publication in Life Science Alliance. Congratulations on this interesting work.

The final published version of your manuscript will be deposited by us to PubMed Central upon publication.

Your manuscript will now progress through copyediting and proofing. At the proofing stage, we request you to complete information for primers in Table S5 (information for RNU6-6P in Table S5 as per your description in methods, line 713).

It is journal policy that authors provide original data upon request.

Your article will publish open access upon publication under a CC-BY license.

Reviews, decision letters, and point-by-point responses associated with peer-review at Life Science Alliance will be published, alongside the manuscript. If you do want to opt out of having the reviewer reports and your point-by-point responses displayed, please let us know immediately.

IMPORTANT: If you will be unreachable at any time, please provide us with the email address of an alternate author. Failure to respond to routine queries may lead to unavoidable delays in publication.

Scheduling details will be available from our production department. You will receive proofs shortly before the publication date. Only essential corrections can be made at the proof stage so if there are any minor final changes you wish to make to the manuscript, please let the journal office know now.

DISTRIBUTION OF MATERIALS:
Authors are required to distribute freely any materials used in experiments published in Life Science Alliance. Authors are encouraged to deposit materials used in their studies to the appropriate repositories for distribution to researchers.

You can contact the journal office with any questions, contact@life-science-alliance.org

Again, congratulations on a very nice paper. I hope you found the review process to be constructive and are pleased with how the manuscript was handled editorially. We look forward to future exciting submissions from your lab.

Sincerely,

Sarita Hebbar, PhD
Scientific Editor
Life Science Alliance
http://www.lsajournal.org
